# Desired Alteration of Protein Affinities: Competitive Selection of Protein Variants Using Yeast Signal Transduction Machinery

**DOI:** 10.1371/journal.pone.0108229

**Published:** 2014-09-22

**Authors:** Misato Kaishima, Nobuo Fukuda, Jun Ishii, Akihiko Kondo

**Affiliations:** 1 Department of Chemical Science and Engineering, Graduate School of Engineering, Kobe University, Kobe, Japan; 2 Organization of Advanced Science and Technology, Kobe University, Kobe, Japan; University of South Florida College of Medicine, United States of America

## Abstract

Molecules that can control protein-protein interactions (PPIs) have recently drawn attention as new drug pipeline compounds. Here, we report a technique to screen desirable affinity-altered (affinity-enhanced and affinity-attenuated) protein variants. We previously constructed a screening system based on a target protein fused to a mutated G-protein γ subunit (Gγ_cyto_) lacking membrane localization ability. This ability, required for signal transmission, is restored by recruiting Gγ_cyto_ into the membrane only when the target protein interacts with an artificially membrane-anchored candidate protein, thereby allowing interacting partners (Gγ recruitment system) to be searched and identified. In the present study, the Gγ recruitment system was altered by integrating the cytosolic expression of a third protein as a competitor to set a desirable affinity threshold. This enabled the reliable selection of both affinity-enhanced and affinity-attenuated protein variants. The presented approach may facilitate the development of therapeutic proteins that allow the control of PPIs.

## Introduction

All biological processes require the control of protein activity, and especially the control of protein-protein interactions (PPIs) [1]. The selection and screening of PPIs has therefore been important for extending our fundamental understanding of biological protein interaction networks and protein functions. Innovative methodologies for identifying PPIs have rapidly grown in all biological fields, in particular in the areas of selection and screening [2].

Recently, molecules that can control PPIs have drawn attention as therapeutic targets and as new pipeline compounds because of their potential to manage pathological activities and the pathogenesis of various diseases via signal transduction, transcriptional regulation, and intracellular metabolism [3]–[5]. PPIs are also used in diagnostic applications in medicinal and biological research fields [6], [7]; for example, these fields are making increasing use of antibodies [8], [9], which can recognize target proteins in a specific manner. For all these applications, directed evolution is a powerful technology for producing protein variants with desirable properties that are not found in nature.

Directed evolution is a general term covering several approaches used in protein engineering to alter a wide range of protein functions, such as activity, stability, selectivity, specificity and affinity [10]–[12]. Affinity maturation is one approach especially used for the engineering of protein affinity and the cell surface display approach, such as phage display and bacterial display techniques, is the most traditional *in vitro* methodology for isolating affinity-enhanced variants from a mutated library [13]–[19]. However, the drawback of this technique is that it requires enrichment procedures and multiple rounds of affinity purification and amplification. In addition, affinity maturation can provide disappointing results due to the inability to completely exclude protein variants causing nonspecific binding or unintentional affinities [20], [21].

For resolving these problems, protein-fragment complementation assays using split β-galactosidase [22], split-GFP [23], [24], split-luciferase [25] and others [26]–[28] were developed. These *in vivo* techniques monitor the reassociation of split reporters as indicators for protein affinities, permitting the selective discrimination of protein variants with different affinities [29]. However, the executable size of library to screen protein variants is limited by the throughput of reporter assays. For example, β-galactosidase reporter is basically compatible with plate assays in 96-well or 384-well formats. Although the GFP is a favorable reporter for high-throughput sorting, an expensive instrument flow cytometer and a skillful technique to set the gating area are needed. Additionally, it has great difficulty separating the variants with the close range of affinities due to the individual variability in the fluorescence levels [30], [31]. Therefore, clear-cut and rapid growth selection on agar plates, which can selectively pick the protein variants with intended affinities, would be a more simple, powerful, versatile approach to screen the large-scale library.

**Figure 1:**
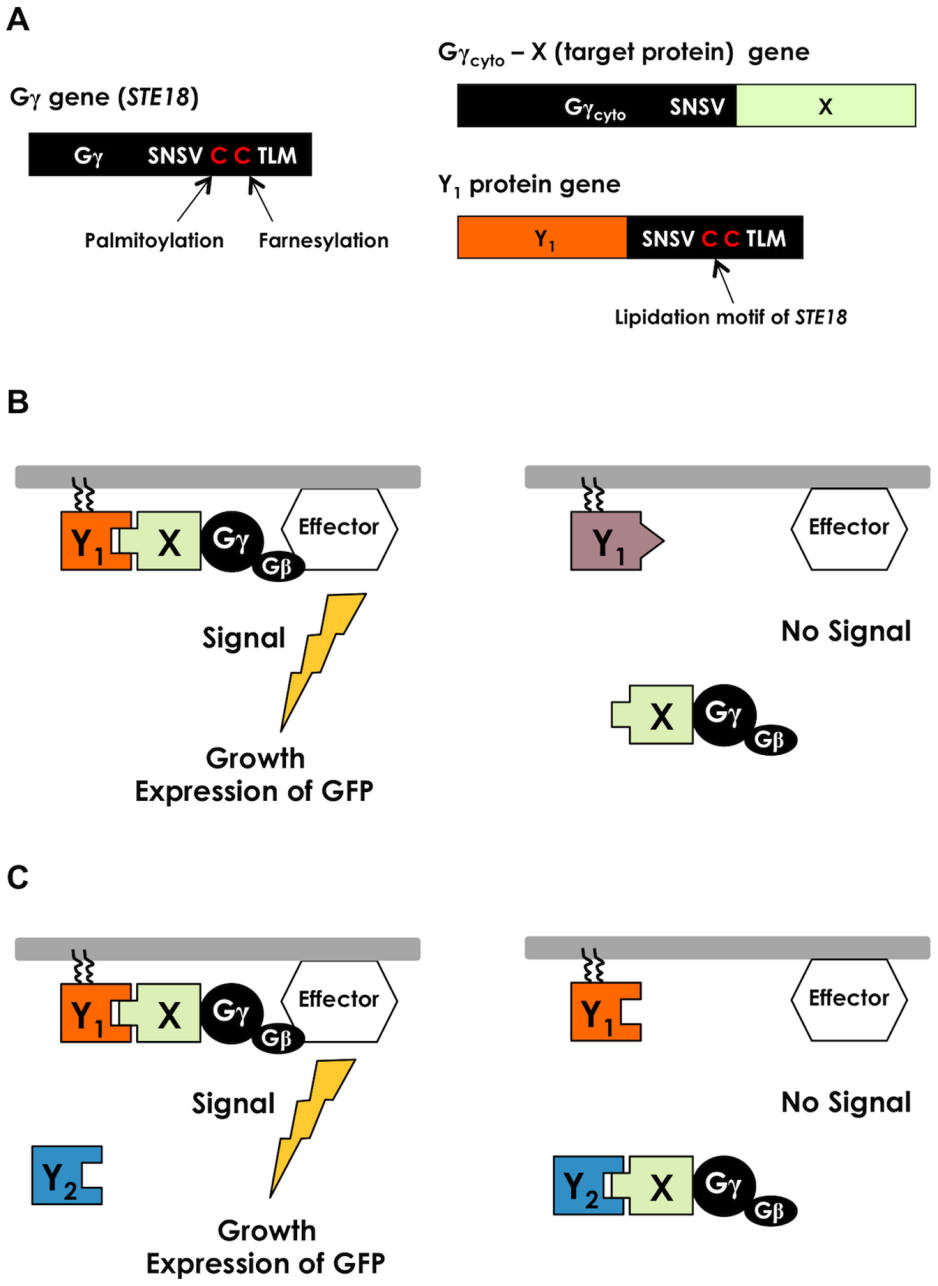
Detection principle behind the competitive Gγ recruitment system. (A) Schematic of the Gγ_cyto_–X and Y_1_ genes. The Gγ_cyto_–X fusion gene is designed to exclude the lipidation motif of the yeast endogenous Gγ (Ste18p). The lipidation motif is artificially attached to the Y_1_ protein to be anchored on the plasma membrane. (B) Schematic outline of previously established Gγ recruitment system to detect PPIs using yeast G-protein signaling. When protein ‘X’ fused to Gγ_cyto_ interacts with protein ‘Y_1_’, the Gβ and Gγ_cyto_ complex (Gβγ_cyto_) migrates to the inner leaflet of the plasma membrane and restores the signaling function. If protein ‘X’ cannot interact with protein ‘Y_1_’, Gβγ_cyto_ is released into the cytosol and signaling is blocked. (C) Schematic outline of the competitive method for creating affinity-altered proteins. Protein “Y_1_” should be anchored on the plasma membrane, whereas “Y_2_” should be expressed in the cytosol. By putting “Y_1_” and “Y_2_” as the parental (known) proteins originally bound to the target ‘X’ or to the candidate variant proteins, respectively, ‘Y_1_’ and ‘Y_2_’ compete to bind against target ‘X’. When “X” has higher affinity for “Y_2_”, G-protein signaling is prevented by sequestration of Gγ_cyto_ from the plasma membrane. When “X” has higher affinity for “Y_1_”, G-protein signaling is transmitted into the yeast cells and invokes the mating process. Thus, affinity-enhanced proteins or affinity-attenuated proteins can be screened in a specific manner. In our system, a transcription assay using the *GFP* reporter gene fused to the signal-responsive *FIG1* gene allows detection of the signaling. Mating growth selection to isolate the methionine- and lysine-prototrophic diploids can also detect the signaling and permits the selective screening of signal-promoted cells.

We previously developed a method, the Gγ recruitment system [32], to detect PPIs based on the fundamental principle that yeast pheromone (mating) signaling requires localization of a complex between guanine nucleotide binding protein (G-protein) β- and γ-subunits (Gβγ) to the inner leaflet of the plasma membrane [33]. In brief, an engineered Gγ mutant (named Gγ_cyto_) lacks a membrane localization sequence (lipidation motif) that is normally expressed in the cytosol. This mutant is prepared in a fused form with a target protein (X) (Gγ_cyto_-X), as shown in **Figure 1A**. Conversely, candidate proteins (Y) in a library are prepared in an attached form with an artificial lipidation motif in order to anchor into the membrane (**Fig. 1A**). If Y has affinity against X, then Gγ_cyto_ complexed with Gβ is recruited onto the membrane and restores the signaling function, thereby permitting the detection of PPIs (**Fig. 1B**). Using the transcription activation or mating process provoked by this signaling, PPI interactive partners can be isolated from the library proteins through the GFP reporter assay or the mating growth selection. Since Gγ_cyto_ localized in the cytosol completely interrupts this signaling, this selection system (Gγ recruitment system) allows very high sensitivity screening [32], [34]. In addition, we have also introduced an attractive approach for screening affinity-enhanced proteins by expressing a binding competitor in the cytosol of the Gγ recruitment system [35]. This approach is applicable for screening affinity-enhanced protein variants selectively. In contrast, a ‘swingable’ screening methodology, which allows alternative screening based on both affinity-enhancement and affinity-attenuation, would allow the selective isolation of protein variants with desired affinities and provide a powerful tool with numerous applications.

Strong PPIs are clearly involved in maintaining the structures of cellular components, but weak interactions are also required for many signaling, transcription networks and cell adhesion phenomena [36]. The ability to fine-tune both strong and weak PPIs flexibly is important because the ability to control PPIs holds great potential for the generation of drugs with reduced side effects and maximum efficacy [37]. The goal of affinity maturation, however, is generally to enhance the protein affinity, while the methodology for massive screening to attenuate the protein affinity scarcely exists. The affinity-attenuated protein variants are indispensable for offering structural information, such as critical amino acid residues for the interactions, to guide the designs of new drugs. Moreover, in recent years, a new field of medicine called multi-target drugs [38] or dirty drugs [39] has attracted attention as a better way of treating complex diseases. To develop such drugs, interaction molecules with weaker affinities than single-target molecules must be developed because these drugs must be able to dissociate from and bind to a variety of different targets [40]–[42].

We here describe the redesign of the previously developed Gγ recruitment system to allow alteration of the affinity of the target protein. This new system allows the mating growth selection to screen selectively both affinity-enhanced and affinity-attenuated protein variants and employs the cytosolic expression of a third protein as a competitor, allowing targeting of a specified affinity threshold. We demonstrate the desirable and reliable selection of both affinity-enhanced and affinity-attenuated protein variants.

## Materials and Methods

### Strains and media

Details regarding *Saccharomyces cerevisiae* BY4741 [43], MC-F1 [44], BY4742 [43] and other recombinant strains used in this study and their genotypes are provided in Tables 1–3. The yeast strains were grown in YPD medium containing 1% (w/v) yeast extract, 2% peptone and 2% glucose, or in SD medium containing 0.67% yeast nitrogen base without amino acids (BD-Diagnostic Systems, Sparks, MD, USA) and 2% glucose. Amino acids and nucleotides (20 mg/L histidine, 60 mg/L leucine, 20 mg/L methionine, or 20 mg/L uracil) were supplemented into SD medium as required by the auxotrophic strains. Agar (2%; w/v) was added to the medium to produce YPD and SD solid media.

**Table 1:** Yeast strains used in this study.

Strain	Relevant feature	Source
BY4741	*MAT*a *his3*Δ*1 ura3*Δ*0 leu2*Δ*0 met15*Δ	[43]
BY4742	*MAT*α *his3*Δ*1 ura3*Δ*0 leu2*Δ*0 lys2*Δ*0*	[43]
MC-F1	BY4741 *fig1*::*FIG1-EGFP*	[44]
BFG2118	MC-F1 *ste18*Δ::*kanMX4 his3*Δ::*URA3-P_STE18_-Gγ_cyto_-Fc*	[32]

**Table 2:** Yeast strains expressing target X (Fc) and parental Y_2_ (Z variants) in the cytosol.

Strain	Relevant feature	Source
BFG2118-ZZcyto	MC-F1 *ste18*Δ::*kanMX4 his3*Δ::*URA3-P_STE18_-Gγ_cyto_-Fc P_HOP2_*::*LEU2-P_PGK_-ZZ_cyto_-P_HOP2_*	Present study
BFG2118-ZWTcyto	MC-F1 *ste18*Δ::*kanMX4 his3*Δ::*URA3-P_STE18_-Gγ_cyto_-Fc P_HOP2_*::*LEU2-P_PGK_-Z_WT,cyto_-P_HOP2_*	Present study
BFG2118-ZK35Acyto	MC-F1 *ste18*Δ::*kanMX4 his3*Δ::*URA3-P_STE18_-Gγ_cyto_-Fc P_HOP2_*::*LEU2-P_PGK_-Z_K35A,cyto_-P_HOP2_*	Present study
BFG2118-ZI31Acyto	MC-F1 *ste18*Δ::*kanMX4 his3*Δ::*URA3-P_STE18_-Gγ_cyto_-Fc P_HOP2_*::*LEU2-P_PGK_-Z _I31A,cyto_-P_HOP2_*	Present study

[For testing the affinity-enhancement system].

**Table 3:** Yeast strains expressing target X (Fc) and parental Y_1_ (Z variants) on the membrane.

Strain	Relevant feature	Source
BZFG2118	MC-F1 *ste18*Δ::*kanMX4-P_PGK1_-ZZ_mem_ his3*Δ::*URA3-P_STE18_-Gγ_cyto_-Fc*	[32]
BFG2Z18-WT	MC-F1 *ste18*Δ::*kanMX4-P_PGK1_-Z_WT,mem_ his3*Δ::*URA3-P_STE18_-Gγ_cyto_-Fc*	[32]
BFG2Z18-K35A	MC-F1 *ste18*Δ::*kanMX4-P_PGK1_-Z_K35A,mem_ his3*Δ::*URA3-P_STE18_-Gγ_cyto_-Fc*	[32]
BFG2Z18-I31A	MC-F1 *ste18Δ::kanMX4-P_PGK_-Z_I31A,mem_ his3*Δ::*URA3-P_STE18_-Gγ_cyto_-Fc*	[32]

[For testing the affinity-attenuation system].

### Construction of plasmids

All plasmids and primers used in this study are listed in Table S1 and Table S2. Plasmids used for the expression of the Z variants (ZZ, Z_WT_, Z_K35A_, Z_I31A_ and Z_955_) [45]–[49] as the library protein on the plasma membrane were constructed as follows. The fragments encoding Z variants with lipidation motifs were amplified from pUMGPT-ZZK, pUMGPT-ZK-WT, pUMGPT-ZK-K35A, pUMGPT-ZK-I31A [32] and pUMGPT-PGKZ955 [*unpublished plasmid; Z_WT_ gene has been replaced by Z_955_ gene in pUMGPT-ZK-WT*] using primer 1 and primer 2, and inserted into the *Sal*I-*Bam*HI sites of the autonomous replication plasmid pGK413 [50], yielding plasmids pGK-HsZZm, pGK-HsZm, pGK-HsZK35Am, pGK-HsZI31Am and pGK-HsZ955m, respectively.

Plasmids used for the expression of the Z variants (ZZ, Z_WT_, Z_K35A_, Z_I31A_ and Z_955_) as the library protein in the cytosol were constructed as follows. The fragments encoding Z variants were amplified from pGK-HsZZm, pGK-HsZm, pGK-HsZK35Am, pGK-HsZI31Am and pGK-HsZ955m using primer 3 and primer 4, and inserted into the *Sal*I-*Bam*HI sites of the autonomous replication plasmid pGK415 [50], yielding plasmids pGK-LsZZc, pGK-LsZWTc, pGK-LsZK35Ac, pGK-LsZI31Ac and pGK-LsZ955c, respectively.

Plasmids used for the integration of the DNA cassettes for expressing Z variants (ZZ and Z_I31A_) upstream of the *HOP2* gene (*P_HOP2_*: *HOP2* promoter region) on the yeast chromosome as the competitor in the cytosol were constructed as follows. The fragments encoding Z variants were amplified from pGK-LsZZc and pGK-LsZI35Ac using primer 3 and primer 4 and inserted into the *Sal*I-*Bam*HI sites of pLMZ-WT-H [35], yielding plasmids pLMZ-ZZ-H and pLMZ-I31A-H, respectively.

Plasmids used for screening of affinity-enhanced proteins and for first step screening of affinity-attenuated proteins (to express the library protein on the plasma membrane) were constructed as follows. The fragments encoding *PGK1* terminator with lipidation motif was amplified from pGK413 [50] using primer 5 and primer 6, and inserted into the *Xma*I-*Not*I sites of the autonomous replication plasmid pGK413 [50], yielding plasmid pGK413-Ste18C. The fragments encoding Z variants were amplified from pGK-LsZWTc, pGK-LsZK35Ac, pGK-LsZI31Ac and pGK-LsZ955c using primer 7 and primer 8, and inserted into the *Sal*I-*Bam*HI sites of pGK413-Ste18C, yielding plasmids pGK413-ZWTmem, pGK413-ZK35Amem, pGK413-ZI31Amem and pGK413-Z955mem, respectively.

Plasmids used for second step screening of affinity-attenuated proteins (to transfer the coding sequences of screened Z variants and express the library protein in the cytosol) were constructed as follows. The fragments encoding *PGK1* terminator with stop codon (TAA) was amplified from pGK415 [50] using primer 9 and primer 6, and inserted into the *Bam*HI-*Not*I sites of the autonomous replication plasmid pGK415 [50], yielding plasmid pGK415-TAA.

Plasmids used for confirming that the lipidated candidate protein (Y_1_) localized to the inner leaflet of the plasma membrane were constructed as follows. The fragment encoding EGFP was amplified from pGK416-EGFP [50] using primer 10 and primer 11, and inserted into the *Sal*I-*Bam*HI sites of the autonomous replication plasmid pGK413 [50], yielding plasmid pGK413-EGFP-N. The fragment encoding Z_WT_ with lipidation motif was amplified from pGK-HsZm using primer 12 and primer 13, and inserted into the *Bam*HI-*Xma*I sites of the autonomous replication plasmid pGK416-EGFP-N, yielding plasmid pGK413-EGFP-ZWTmem.

### Construction of yeast strains

All strains used in this study are listed in Tables 1-3, and all transformants used in this study are listed in Table S3 and Table S4. Integration of the DNA cassettes for expressing Z variants (ZZ, Z_WT_, Z_K35A_ and Z_I31A_) as the competitor in the cytosol was achieved as follows. The DNA fragments containing *LEU2-PGK5’-Z*-*PGK3’-P_HOP2_* (*PGK5’*, *PGK1* promoter; *PGK3’*, *PGK1* terminator) were amplified from pLMZ-ZZ-H, pLMZ-WT-H, pLMZ-K35A-H [35] and pLMZ-I31A-H using primers 14 (containing the homologous regions of *P_HOP2_* upstream) and 15. The amplified DNA fragments were used to transform BFG2118 [32] using the lithium acetate method [51]. The transformants were selected on SD-Leu, -Ura plate (SD solid medium without leucine and uracil, but containing histidine and methionine), to yield BFG2118-ZZcyto, BFG2118-ZWTcyto, BFG2118-ZK35Acyto and BFG2118-ZI31Acyto (**Table 2**). All transformants were obtained by introducing the plasmids into the yeast strains using the lithium acetate method (**Table S3, Table S4 and Table S7**).

### Fluorescence imaging by confocal laser scanning microscopy

The pGK413-EGFP-ZWTmem-introduced BFG2118-ZK35Acyto yeast, which expressed the GFP-fused Z_WT_ with an artificial lipidation motif, was grown in SD-His, -Leu, -Ura medium at 30°C for 18 hours. The cultured cells were washed and resuspended in distilled water to yield an optical density of 40 at 600 nm (OD_600_ of 40). The cell suspensions were observed with a LSM 5 PASCAL confocal laser scanning microscope (Carl Zeiss, Oberkochen, Germany). Fluorescence image was acquired using the 488 nm line of an argon laser for excitation and a 505-nm band pass filter for emission.

### GFP reporter expression analysis

GFP reporter expression analysis basically followed previous methods [32], [35] with some modifications. The engineered yeast **a**-cells were grown in 5 mL of SD-His, -Leu, -Ura media (for affinity enhancement) or SD-Leu, -Ura media (for affinity attenuation) at 30°C overnight. The cultured cells were inoculated into fresh 2 mL of SD-His, -Leu, -Ura or SD-Leu, -Ura media containing 5 µM α-factor (Zymo Research, Orange, CA, USA) to give an initial OD_600_ of 0.1. Then, the expression of *FIG1-EGFP* fusion reporter gene was stimulated by growing at 30°C for 6 hours.

Fluorescence intensities of cultured cells were measured using a BD FACSCanto II flow cytometer equipped with a 488-nm blue laser (BD Biosciences, San Jose, CA, USA) [52]. The EGFP fluorescence signal was collected through a 530/30-nm band-pass filter. The mean of fluorescence intensity was defined as the GFP-A mean of 10,000 cells. The data were analyzed using BD FACSDiva software (version 5.0, BD Biosciences).

After washing the cultured cells three times, the cells were resuspended in distilled water and observed under a BIOREVO BZ-9000 fluorescence microscope (KEYENCE, Osaka, Japan). Green fluorescence images were acquired with a 470/40 band-pass filter for excitation and a 535/50 band-pass filter for emission.

### Mating growth spotting assay

The mating growth spotting assay basically followed a previous method [35] with some modifications. Each engineered yeast **a**-cell strain was grown in 5 mL of SD-His, -Leu, -Ura medium (for affinity enhancement) or SD-Leu, -Ura medium (for affinity attenuation) at 30°C overnight, and then cultivated in 5 mL of YPD medium with the mating partner, BY4742 α-cell [43], at 30°C for 3 hours. The initial OD_600_ of each haploid cell strain was set at 0.1. After cultivation, the yeast cells were harvested, washed, and resuspended in distilled water. To quantify the mating ability of each strain, a dilution series of each yeast cell suspension was prepared (OD_600_ = 1.0, 0.1, 0.01, 0.001 and 0.0001), then 30 µL of each dilution was spotted on a diploid-selective SD plate (lacking methionine, lysine, histidine, leucine and uracil; for affinity enhancement) or SD+His plate (lacking methionine, lysine, leucine and uracil; for affinity attenuation).

### Screening of affinity-enhanced proteins from model libraries

**Figure 2:**
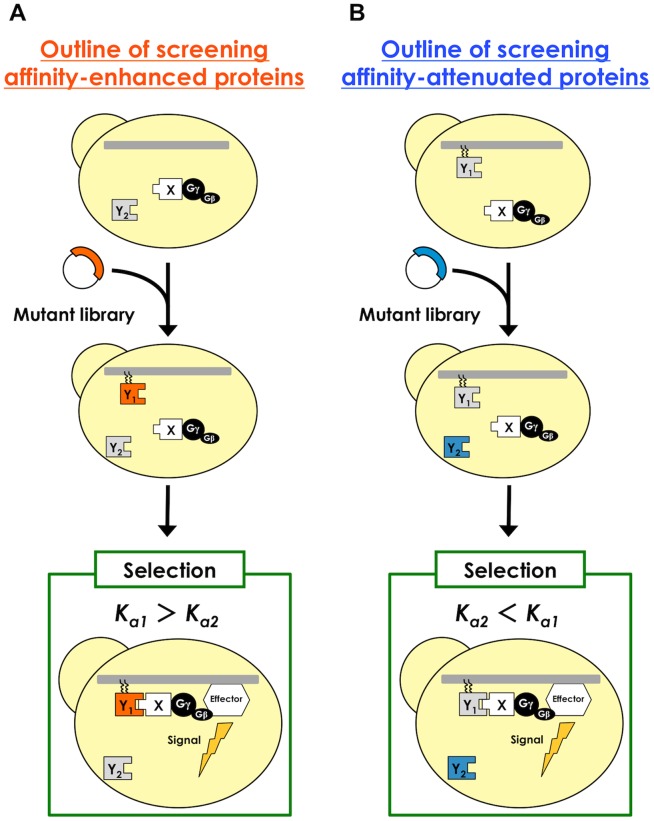
Flow diagram of the selection principle behind the competitive Gγ recruitment system. (A) Outline for the selection of desirable affinity-enhanced proteins. The yeast stain expressing target protein ‘X’ fused with the Gγ mutant (Gγ_cyto_-X) and the competitor protein ‘Y2’ in the cytosol is transformed with the plasmid expressing the mutant library on the inner leaflet of the plasma membrane (Y_1_). Preferential binding of “X” to “Y_1_” (*K_a1_*>*K_a2_*) restores the signaling function and permits the selective screening of affinity-enhanced proteins. (B) Outline for screening affinity-attenuated proteins. The yeast stain expressing a target protein ‘X’ fused with the Gγ mutant (Gγ_cyto_-X) and a competitor protein ‘Y1’ on the inner leaflet of the plasma membrane is transformed with the plasmid expressing the mutant library in the cytosol (Y_2_). Preferential binding of “X” to “Y_1_” (*K_a2_*<*K_a1_*) restores the signaling function and permits the selective screening of affinity-attenuated proteins.

Flow diagram for screening of affinity-enhanced proteins is shown in Figure 2A. Plasmid libraries (to express Z variants as membrane ‘Y_1_’) with varied compositions were prepared by mixing the target plasmid (pGK413-ZWTmem) and the control plasmids (pGK413-ZK35Amem, pGK413-ZI31Amem, pGK413-Z955mem and pGK413) (**Table S5**) and introduced into yeast BFG2118-ZK35Acyto (to express competitive Z_K35A_ as cytosolic ‘Y_2_’) by the lithium acetate method [51] with some modifications. Briefly, overnight cultured cells in YPD media were washed with distilled water, pelleted, and then resuspended in 1500 µl of TE/LiAc solution (10 mM Tris-HCl, 1 mM ethylenediaminetetraacetate (EDTA) and 100 mM lithium acetate; pH 7.5). Aliquots of 100 µl of yeast competent cells were mixed with 20 µg of plasmid libraries, 200 µg heat-denatured carrier DNA (Takara/Clontech Laboratories, Shiga, Japan) and 600 µl of PEG/LiAc solution (10 mM Tris-HCl, 1 mM EDTA, 100 mM lithium acetate and 40% polyethylene glycol 4000 (PEG4000)) and incubated at 30°C for 30 minutes. After adding 70 µl of dimethyl sulfoxide (DMSO) (Nakarai Tesque, Kyoto, Japan), the cell suspensions were incubated at 42°C for 15 minutes, transferred into 20 mL of SD-His, -Leu, -Ura media, and then cultured at 30°C for 1 day. The cultured cells were inoculated into 100 mL of YPD media to set the initial OD_600_ of each haploid cell at 0.1 with the mating partner BY4742, and cultivated at 30°C for 6 hours (for library No. 1 and No. 2) or 9 hours (for library No. 3 and No. 4) (**Table S5**). After cultivation, yeast cells were harvested by centrifugation (3000 g, 5 min), washed and resuspended with distilled water, and then spread on diploid-selective SD plates (lacking methionine, lysine, histidine, leucine and uracil). After incubation at 30°C for 2 days, 20 colonies were picked up and analyzed to determine the screened candidate proteins by direct colony PCR using primer 16 and primer 17 and sequencing of the amplified fragments. The final ratio of target plasmid was determined by the number of colonies retaining the target plasmid (Z_WT_) divided by the number of picked colonies (20 colonies).

### Screening of affinity-attenuated proteins from model libraries

Flow diagram for screening of affinity-attenuated proteins is shown in Figure S1. For first step screening, plasmid libraries (to express Z variants as membrane ‘Y_1_’) with varied compositions were prepared by mixing the target plasmid (pGK413-ZK35Amem and pGK413-ZI31Amem) and the control plasmids (pGK413-ZWTmem, pGK413-Z955mem and pGK413) (**Table S5**) and introduced into yeast BFG2118 [32] (lacking the expression of competitive proteins) by the same procedures described in the previous section. The transfomants were cultured in SD-His, -Ura media for 1 day, inoculated into YPD media with BY4742, and cultivated for 6 hours. After washing, the cells were spread on diploid-selective SD+Leu plates (lacking methionine, lysine, histidine and uracil) and incubated for 2 days. One-hundred colonies were picked up and the fragments containing genes encoding the screened candidate proteins were amplified individually by direct colony PCR using primer 16 and primer 17 and the sequences of screened candidates were analyzed. Then, the amplified fragments were mixed and digested with *Sal*I and *Bam*HI and inserted into the *Sal*I-*Bam*HI sites of the autonomous replication plasmid pGK415-TAA, yielding the plasmid pGK415-Library for the second step screening.

For second step screening, the obtained pGK415-Library (to express Z variants as cytosolic ‘Y_2_’) were introduced into yeast BFG2Z18-WT [32] (to express competitive Z_WT_ as membrane ‘Y_1_’) by the same procedures described in the previous section. The transfomants were cultured in SD-Leu, -Ura media for 1 day, inoculated into YPD media with BY4742, and cultivated for 1 hour. After washing, the cells were spread on diploid-selective SD+His plates (lacking methionine, lysine, leucine and uracil) and incubated for 2 days. Twenty colonies were picked up and analyzed to determine the screened candidate proteins by direct colony PCR using primer 18 and primer 19 and sequencing of the amplified fragments. The final ratio of target plasmids were determined by the number of colonies retaining the target plasmids (Z_K35A_ and Z_I31A_) divided by the number of picked colonies (20 colonies).

For an additional second round of second step screening, one-hundred colonies were picked up from the plate after the second step screening and the retaining plasmids were collectively extracted using the SpeedPrep Yeast Plasmid Isolation kit (DualSytems Biotech AG, Schlieren, Switzerland) with some modifications. The incubation time for Lytic agent was prolonged up to overnight and the MonoFas Spin column (GL Science, Tokyo, Japan) was alternatively used for the plasmid recovery. The extracted plasmid mixture was introduced into yeast BFG2Z18-WT and then the same screening procedure described above (in the second step screening section) was performed.

## Results

### General strategy used

First, target ‘X’ is expressed as a fusion protein with the Gγ mutant (Gγ_cyto_-X), as shown in **Figure 1A and B**. Then, another protein, ‘Y_1_’, is expressed as an anchored protein on the inner leaflet of the plasma membrane (**Fig. 1A and B**). A third protein, ‘Y_2_’, is expressed in the cytosol (**Fig. 1C**). By placing ‘Y_1_’ and ‘Y_2_’ either as the parental (known) proteins originally bound to target ‘X’, or as the candidate variant proteins, ‘Y_1_’ and ‘Y_2_’ compete to bind against target ‘X’. If ‘Y_1_’ binds to ‘X’ preferentially (rather than to ‘Y_2_’), Gγ_cyto_ should gravitate toward the plasma membrane and restore the signaling function (**Fig. 1C**). On the basis of this tenet, we devised an adjustable method for screening protein variants for desirable affinities, as follows.

To isolate affinity-enhanced proteins, the parental protein known to bind to target ‘X’ is expressed as a cytosolic ‘Y_2_’ protein (**Fig. 2A**). When introducing the mutant library containing the candidates as membrane-anchored ‘Y_1_’ proteins into the yeast, only the transformants that express the candidate proteins (Y_1_) exhibiting stronger affinity against target ‘X’ than the parental protein (Y_2_) can transduce the signal inside the cells (**Fig. 2A**). Conversely, to isolate the affinity-attenuated proteins, the parental protein bound to target ‘X’ and the candidate mutant library are expressed as membrane-anchored ‘Y_1_’ and cytosolic ‘Y_2_’, respectively; in this instance, only the transformants that express the candidate proteins (Y_2_) exhibiting lower affinity against target ‘X’ than the parental protein (Y_1_) can activate signal transduction in the yeast (**Fig. 2B**). Thus, our competitive approach should selectively exclude unsolicited protein variants and preserve only the desirable protein variants by using signal transmission as the indicator.

**Figure 3:**
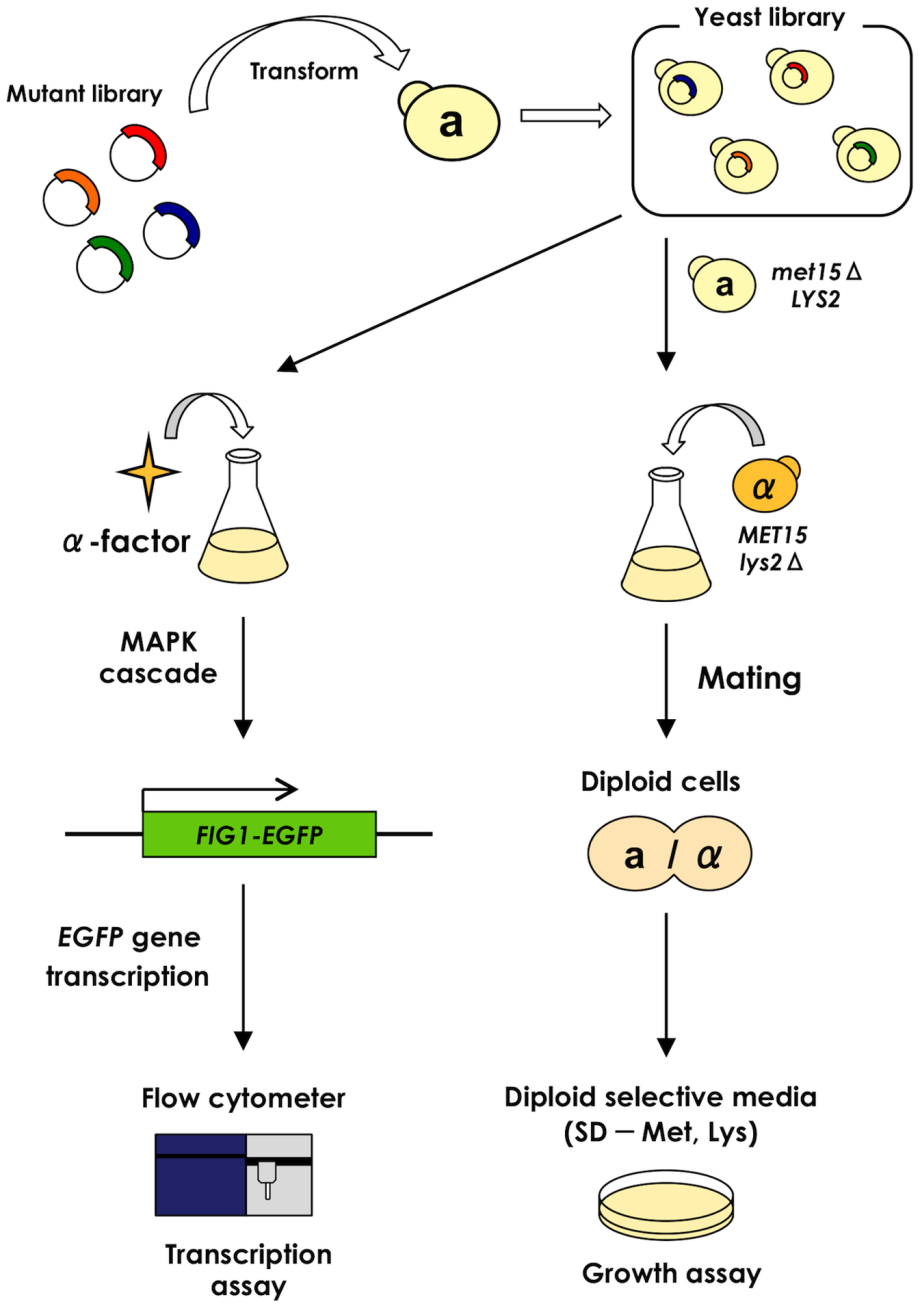
Flow diagram of the screening procedure for the competitive Gγ recruitment system. Two selection methods are available to screen affinity-altered proteins. One is to use the *GFP* reporter gene. When target candidate proteins are expressed in the yeast cells and interact with each other, they induce *GFP* expression and are isolated by flow cytometry. Another method is to use yeast mating detected using a growth assay. When target candidate proteins are expressed in yeast cells, they restore mating ability and grow on the diploid-selective medium.

We employed yeast BY4741 (haploid **a**-cell) with **a**-specific methionine auxotrophy [43] as a parent to construct our recombinant strains (**Table 1**). Mating with wild-type BY4742 haploid α-specific lysine-auxotroph [43] allowed convenient screening of the signal-promoted **a**-cells using the selective growth of diploid cells on solid media lacking methionine and lysine (**Fig. 3**). The recombinant **a**-type strain was additionally engineered to induce the transcription of the *GFP* reporter gene in response to the mating signal (Table 1; MC-F1 derivative strains), thereby allowing fluorescence to be used as the output signal [44] (**Fig. 3**).

### Validation of the concept for screening affinity-enhanced proteins

To demonstrate our strategy for selecting affinity-enhanced proteins specifically (**Fig. 2A**), we used the Fc portion of human immunoglobulin G (IgG) as the target protein ‘X’. The candidate ‘Y_1_’ proteins or the known ‘Y_2_’ parental proteins were constructed from the Z domain derived from *Staphylococcus aureus* protein A (Z_WT_) to yield several Z variants (ZZ, Z_K35A_, Z_I31A_ and Z_955_) with different affinities against the Fc portion (**Table 4**) [45]–[49]. In order to make this method easily accessible, the DNA cassettes for expressing the candidate ‘Y_1_’ proteins were introduced using autonomous replication plasmids (**Table S1**), whereas those for expressing the target protein ‘X’ and known ‘Y_2_’ parental proteins were stably integrated into the yeast chromosome (**Table 2** **and Table S6**).

**Table 4:** List of affinity constants of the Z variants for the Fc part of human IgG.

Type of Z domain	Affinity constant for the Fc part of human IgG [M^−1^]	Source
ZZ	6.8×10^8^	[45]
Z_WT_	5.9×10^7^	[45]
Z_K35A_	4.6×10^6^	[45]
Z_I31A_	8.0×10^3^	[45]
Z_955_	None	[48]

**Figure 4:**
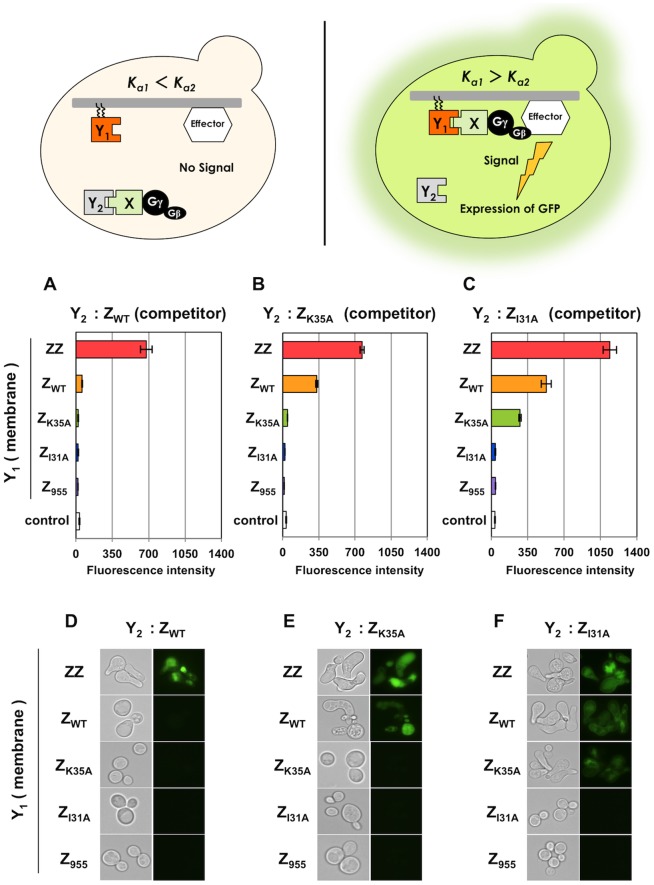
*GFP* transcription assays to test the selection of affinity-enhanced proteins. (A–C) Flow cytometry analyses. Fluorescence intensities of the engineered strains expressing cytosolic Z_WT_ (A), Z_K35A_ (B) and Z_I31A_ (C) as the parental ‘Y_2_’ proteins. White bars indicate control yeast strains without the expression of ‘Y_1_’ (transformed with pGK413 mock vector). (D–F) Fluorescence microscope observations. Fluorescence micrographs of the engineered strains expressing cytosolic Z_WT_ (D), Z_K35A_ (E) and Z_I31A_ (F) as the parental ‘Y_2_’ proteins. Five Z variants (ZZ, Z_WT_, Z_K35A_, Z_I31A_ and Z_955_) were expressed as the membrane-anchored ‘Y_1_’ candidate proteins in each strain. To investigate transmission of the signal, 5 µM of α-factor was used for each strain. Standard errors of three independent experiments are shown.

**Figure 5:**
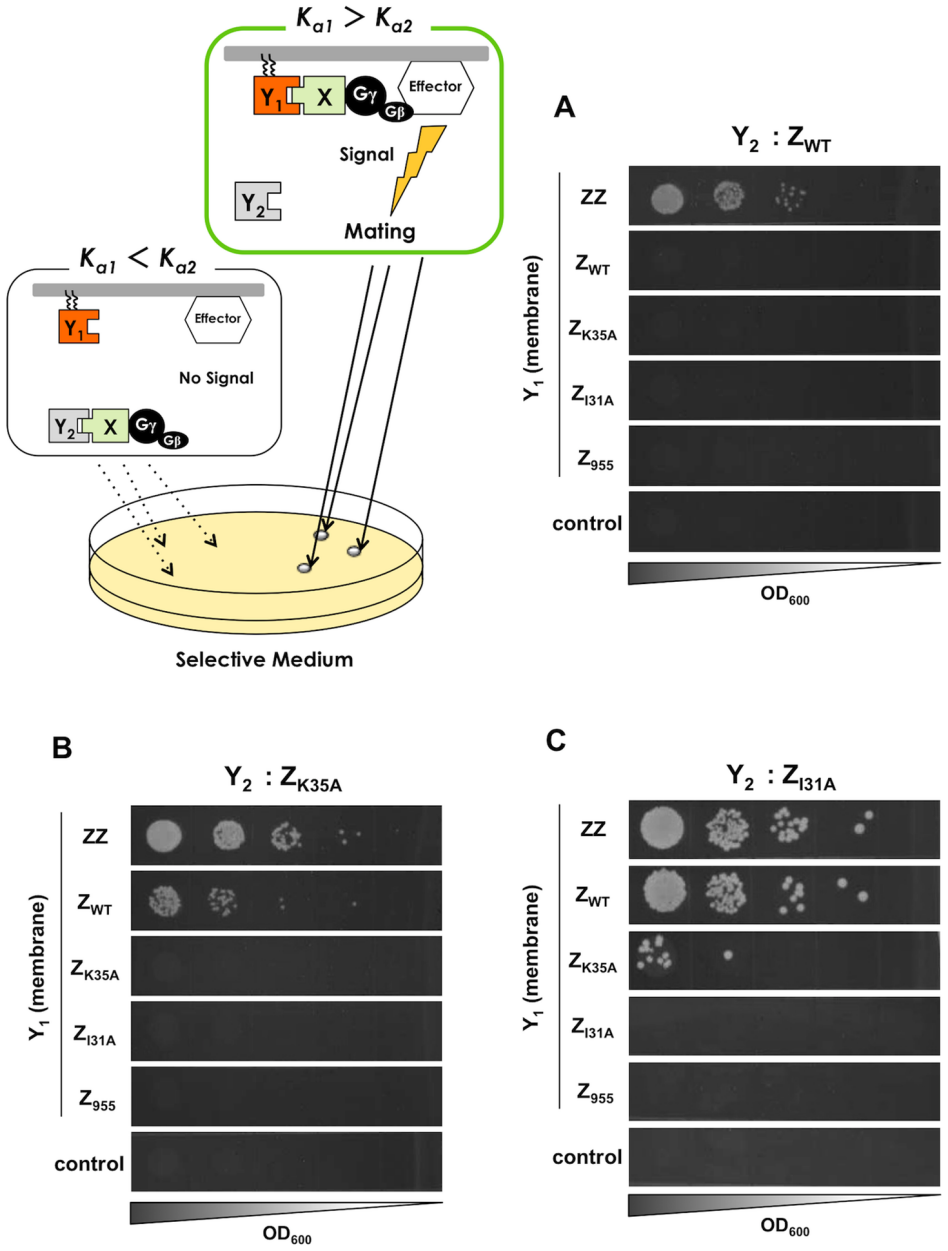
Mating growth selection to test the screening of affinity-enhanced proteins. The signal-promoted cells were isolated as methionine- and lysine-prototrophic diploids. Growth of the engineered strains expressing cytosolic Z_WT_ (A), Z_K35A_ (B) and Z_I31A_ (C) as the parental ‘Y_2_’ proteins. Five Z variants (ZZ, Z_WT_, Z_K35A_, Z_I31A_ and Z_955_) were expressed as the membrane-anchored ‘Y_1_’ candidate proteins in each strain. Control indicates the yeast strains without the expression of ‘Y_1_’ (transformed with pGK413 mock vector). BY4742 was used as the mating partner.

First, we selected Z_WT_ as the parental ‘Y_2_’ protein and expressed five different Z variants (ZZ, Z_WT_, Z_K35A_, Z_I31A_ and Z_955_) as the candidate ‘Y_1_’ protein in place of the mutant library (**Table S3**). Flow cytometric analyses and fluorescence microscopic observations were conducted after incubation in medium containing the **a**-cell-specific mating pheromone (α-factor). The engineered yeast strain expressing the membrane-anchored ZZ as the candidate ‘Y_1_’ specifically induced the transcription of the *GFP* reporter gene (**Fig. 4A and D**). This shows that the target-fused Gγ_cyto_-Fc protein permitted signal transmission only by using the ZZ protein as an intermediary in the presence of the parental Z_WT_ protein in the cytosol, and not the other membrane-bound variants. These results suggest that the competitive expression of the cytosolic ‘Y_2_’ parental protein could specifically discern the affinity-enhanced proteins. Mating selection on diploid-selective medium followed the transcription assays (**Fig. 5A**).

Other variants (Z_K35A_ and Z_I31A_) expressed as cytosolic ‘Y_2_’ proteins (**Table S3**) showed similar trends as Z_WT_, verifying that the competitive approach using Gγ_cyto_ can effectively isolate affinity-enhanced proteins (**Figs. 4B, E** **and** **5B**) (**Figs. 4C, F** **and** **5C**). Supportively, it was confirmed that the lipidated candidate protein (Y_1_) fused with EGFP (EGFP-Z_WT_-Ste18C) (**Table S1**) successfully localized to the inner leaflet of the plasma membrane in the engineered **a**-type yeast cells (**Fig. S1**).

To further test the capabilities of our system for screening affinity-enhanced proteins, Z_K35A_ was selected as the model of parental ‘Y_2_’ protein. Plasmid libraries with four different compositions were prepared to express the Z variants as the candidate ‘Y_1_’ proteins on the membrane (**Table S5**). The prepared plasmid libraries were introduced into the engineered **a**-type yeast cells, in which the Z_K35A_ was competitively expressed as cytosolic ‘Y_2_’ (BFG2118-ZK35Acyto) (**Table 2**). The obtained four yeast **a**-cell libraries were respectively co-cultured with α-type BY4742 wild-type yeast cells and mating selection on diploid-selective media was performed. As shown in **Table 5**, the accuracy rates to yield the target protein (Z_WT,mem_) have been maintained at 75% even when the library with 0.1% frequency of target plasmid was used, while they gradually decreased as the initial rates had become smaller. It was further notable that our affinity-enhancement system enabled to isolate the target plasmid by just one cycle screening procedure.

**Table 5:** Screening efficiency of the affinity-enhancement system form model libraries using growth selection.

No.	Initial ratio of target plasmid (Z_WT_)	Final ratio of target plasmid (Z_WT_)
1	10%	100%
2	1%	90%
3	0.5%	85%
4	0.1%	75%

Thus, the results demonstrate that this approach can exclude unwanted proteins and isolate promising affinity-enhanced candidate proteins specifically, indicating that our method holds promise as a practical tool for screening affinity-enhanced proteins specifically from a mutant library.

### Validation of the concept for screening affinity-attenuated proteins

To validate our hypothesis for selectively sorting affinity-attenuated proteins (**Fig. 2B**), we used Z_WT_, Z_K35A_ and Z_I31A_ as parental ‘Y_1_’ proteins, and the five Z variants as candidate ‘Y_2_’ proteins in the mutant library (**Table S4**). As described above, the Fc portion was used as the target protein ‘X’. The DNA cassettes for expressing the candidate ‘Y_2_’ proteins were introduced using autonomous replication plasmids (**Table S1**), whereas those for expressing the target protein ‘X’ and known ‘Y_1_’ parental proteins were stably integrated into the yeast chromosome (**Table 3** **and Table S6**).

**Figure 6:**
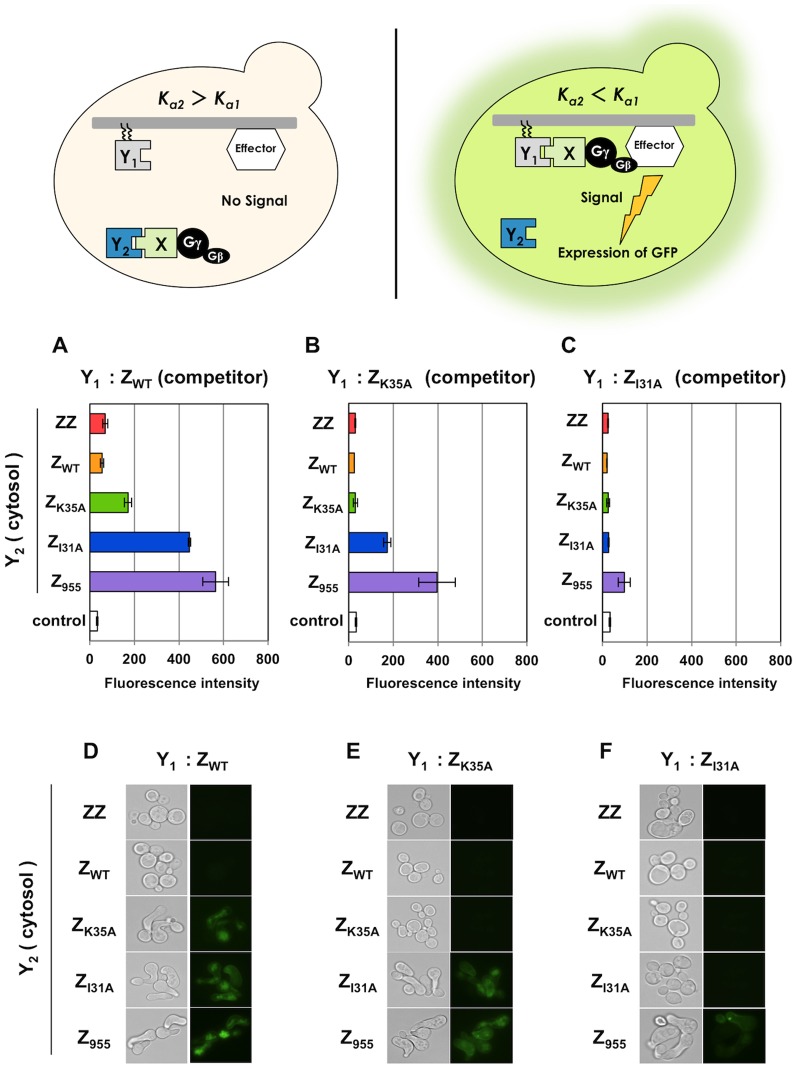
*GFP* transcription assays to test the selection of affinity-attenuated proteins. (A–C) Flow cytometry analyses. Fluorescence intensities of the engineered strains expressing membrane-anchored Z_WT_ (A), Z_K35A_ (B) and Z_I31A_ (C) as the parental ‘Y_1_’ proteins. White bars indicate control yeast strain lacking expression of ‘Y_1_’ and ‘Y_2_’ (transformed with pGK415 mock vector to BFG2118). (D–F) Fluorescence microscope observations. Fluorescence micrographs of the engineered strains expressing membrane-anchored Z_WT_ (D), Z_K35A_ (E) and Z_I31A_ (F) as the parental ‘Y_2_’ proteins. Five Z variants (ZZ, Z_WT_, Z_K35A_, Z_I31A_ and Z_955_) were expressed as the cytosolic ‘Y_2_’ candidate proteins in each strain. To investigate transmission of the signal, 5 µM of α-factor was used for each strain. Standard errors of three independent experiments are shown.

**Figure 7:**
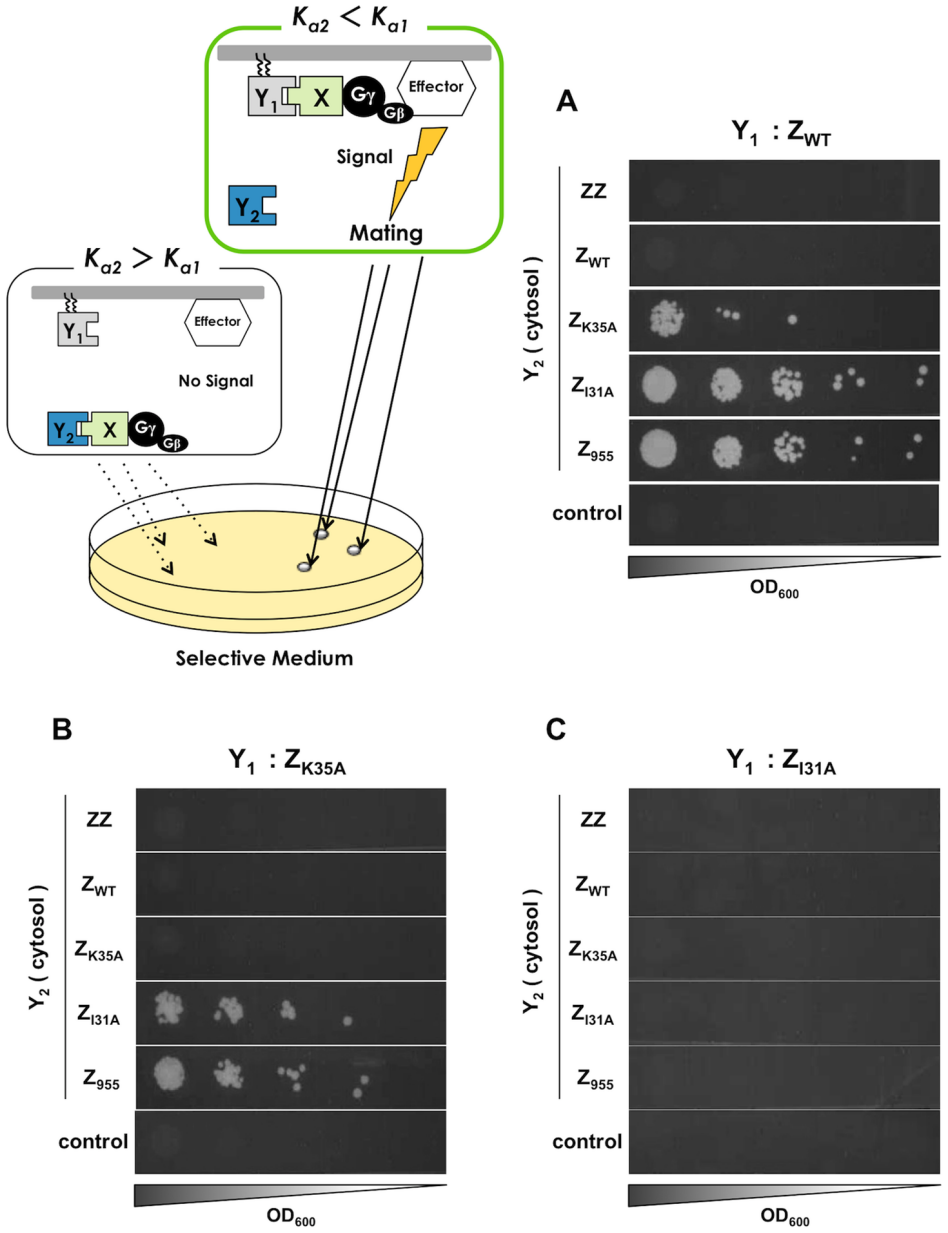
Mating growth selection to test the screening of affinity-attenuated proteins. The signal-promoted cells were isolated following formation of methionine- and lysine-prototrophic diploids. Growth of the engineered strains expressing membrane-anchored Z_WT_ (A), Z_K35A_ (B) and Z_I31A_ (C) as the parental ‘Y_1_’ proteins. Five Z variants (ZZ, Z_WT_, Z_K35A_, Z_I31A_ and Z_955_) were expressed as the cytosolic ‘Y_2_’ candidate proteins in each strain. Control indicates the yeast strain lacking expression of ‘Y_1_’ and ‘Y_2_’ (transformed with pGK415 mock vector to BFG2118). BY4742 was used as the mating partner.

When using Z_WT_ as the membrane-anchored parental protein (**Table S4**), the engineered yeast cells expressing the three cytosolic Z variants with lower affinities than Z_WT_ (Z_K35A_, Z_I31A_ and Z_955_) predictably displayed green fluorescence following incubation in α-factor-containing medium (**Fig. 6A and D**). The *GFP* transcription assays were followed by mating growth selection to isolate the methionine- and lysine-prototrophic diploids (**Fig. 7A**). The engineered yeast strains expressing Z_K35A_ and Z_I31A_ as membrane-anchored parental proteins also provided predictable results (**Figs. 6B, E** **and** **7B**) (**Fig. 6C and F**), although mating selection using Z_I31A_ as Y_1_ provided no colonies (**Fig. 7C**). These results indicate that mating selection using our methodology allows the specific screening of affinity-attenuated candidate proteins, although it has limited ability to detect candidate proteins exhibiting extremely low affinities.

Next, to demonstrate the feasibility of the system for screening affinity attenuated-proteins, we tested a two-step selection (**Fig. S2**). The original Gγ recruitment system (**Fig. 1B**) was used as the first selection to screen all protein mutants with affinity to the target protein, and then the competitive method (**Fig. 2B**) was used as the second selection to narrow down the affinity-attenuated candidate proteins. For the first selection, plasmid libraries with two different compositions were prepared to express the Z variants as the candidate ‘Y_1_’ proteins on the membrane (**Table S5, Fig. S2 and Fig. S3**). The prepared plasmid libraries were introduced into the engineered **a**-type yeast cells lacking the expression of competitive cytosolic proteins (BFG2118) (**Table 1**). The obtained two yeast **a**-cell libraries were respectively co-cultured with α-type BY4742 yeast cells and the mating selection on diploid-selective media was carried out. One-hundred colonies were picked up to amplify the fragments containing the candidate genes and checked whether the false positive candidates lacking affinity to the Fc were omitted in the first selection as expected (data not shown). Then, the fragments corresponding to the gene encoding regions of Z variants were digested from the mixture of fragments and inserted into the pGK415-TAA plasmid (**Table S1**) for cytosolic ‘Y_2_’ expression to screen the affinity-attenuated proteins (**Fig. S2**). The constructed pGK415-Library was introduced into engineered **a**-type yeast cells, in which the Z_WT_ was competitively expressed as membrane ‘Y_1_’ (BFG2Z18-WT) (**Table 3**). The obtained **a**-cell library was co-cultured with BY4742 and the mating selection on diploid-selective media was performed. As shown in **Table 6**, the accuracy rate to yield the target proteins (Z_K35A_ and Z_I31A_) was 95% when the library with 2% frequency of target plasmids was used. Even when using the library with 0.2% target frequency, the yeast cells expressing the target proteins were successfully attained with 55% of accuracy rate (**Table 6**). As the available option to increase the accuracy rate, an additional round of second-step screening was tested. As the result, the accuracy rate for screening of targets was elevated up to 85% (**Table 6**). In addition, when the Z variant genes were directly amplified from the mixture of massive colonies (>1000 colonies) obtained by the first mating selection, target clones were also screened after the second-step selection (data not shown). Thus, we successfully proved that the selective screening of affinity-attenuated proteins by two-step (or three-step) selection was feasible, although the system has still significant scope for improvement.

**Table 6:** Screening efficiency of the affinity-attenuation system form model libraries using growth selection.

No.	Initial ratio of target plasmids (Z_K35A_ + Z_I31A_)	Final ratio of target plasmids		Final ratio of total target plasmids (Z_K35A_ + Z_I31A_)
		Z_K35A_	Z_I31A_	
1	2.0%	80%	15%	95%
2-1*	0.2%	55%	0%	55%
2-2**	0.2%	85%	0%	85%

* “2-1” shows the screening efficiency for No. 2 library after one round of second-step selection.

** “2-2” shows the screening efficiency for No. 2 library after two round of second-step selection.

Finally, we clarified unique aspects of our method. When the tandem fusion form of Z domain (ZZ) was used as the parental protein, both affinity enhancement (**Table S3 and Fig. S4**) and affinity attenuation systems (**Table S4 and Fig. S5**) were accompanied by candidate proteins which exhibited the same affinity as the original protein, ZZ. This indicated that the expression level of ZZ as the cytosolic ‘Y_2_’ was insufficient and needed to be increased. We therefore introduced an additional one- or multi-copy replication plasmid to overexpress ZZ as the ‘Y_2_’ parental protein in the affinity enhancement system, in addition to the chromosomally-integrated *ZZ* gene (**Table S3 and Fig. S6**). Using the multi-copy plasmid, the affinity enhancement system displayed the desired outcome, namely, no activation of the *GFP* transcription (**Fig. S6**). For the affinity attenuation system when expressing ZZ as the parental Y_1_ protein, we introduced a multi-copy plasmid instead of the one-copy plasmid to overexpress ZZ as the ‘Y_2_’ candidate protein (**Table S4 and Fig. S7**) and observed the desired outcome, namely, suppression of *GFP* transcription activation similar to that in the affinity enhancement system (**Fig. S7**). Thus, controlling the expression level of the parental protein is important for robust screening of both affinity-enhanced and affinity-attenuated proteins.

## Discussion

The above results demonstrate the utility of our system for screening affinity-altered protein variants. While various screening studies can be performed using existing screening systems, many, such as phage display or yeast two-hybrid systems, screen protein variants exhibiting any affinity, making it difficult or impossible to screen affinity-altered proteins selectively. To address this issue, we expanded our previously constructed Gγ recruitment system into a competitive-binding system, thereby opening the door to the selective and reliable screening of both affinity-enhanced and affinity-attenuated protein variants.

The competitive approach allows the specific selection of cells expressing affinity-enhanced candidate proteins (**Figs. 4** **,** **5** **, and** **Table 5**), since signal transmission requires the localization of Gγ onto the membrane. Consequently, the inability to localize into the membrane leads to insensitivity to the transmission of background signals [32], [34]. Thus, our system could be useful as an alternate technique for the phage display technology and protein-fragment complementation assays [13]–[19], [22]–[28], which can screen affinity-enhanced protein variants. Because yeast can express a variety of affinity proteins, including full-length antibodies [53] and antibody-like proteins, our system will be easily applicable to a wide range of directed evolution research and be able to screen protein variants with stronger affinities. In addition, for example, a protein scaffold that is a constrained polypeptide consisting of either an α-helix or a β-sheet and one or several binding activity domains [54]–[56] could be used in our system to engineer novel binding proteins. Furthermore, using the original Gγ recruitment system [32] (**Fig. 1B**) in combination with the current system (**Fig. 2A**), it will be possible to make variants of novel protein scaffolds that strongly bind to the target protein.

Our results demonstrate that the competitive approach successfully screens cells expressing the desired affinity-attenuated protein variants (**Figs. 6** **,** **7** **and** **Table 6**). Until recently, there were few practical methods for selectively screening protein variants with weak affinities. In that sense, the current method opens new possibilities for designing multi-target drugs and dirty drugs, and for making drugs with relatively few side effects.

In the attenuated system, extremely low affinity, as in the case of mating selection with Z_I31A_ parental protein (**Fig. 7C**), cannot be detected. Due to the very low affinity between Z_I31A_ and the Fc region (8.0×10^3^ M^−1^), the signaling level was likely insufficient for the mating process. This affinity (8.0×10^3^ M^−1^) seems to be less than a lower limit of the mating selection, although it is unlikely that a protein mutant exhibiting such extremely low association constant would be required. It is important to note that this method detects both affinity-attenuated and approximately zero-affinity proteins such as Z_955_ (**Figs. 6** **and** **7**). Therefore, care must be taken to screen the affinity-attenuated proteins specifically. For instance, a two-step selection would be advisable as shown in **Fig. S2**. Specifically, the original Gγ recruitment system (**Fig. 1B**) was used as the first selection to screen for protein mutants with affinity to the target protein, while the competitive method (**Fig. 2B**) was used as the second selection to narrow down the affinity-attenuated candidate proteins. Additionally, we used Z_955_ expression plasmid and mock vector as alternatives to a significant part of incomplete error-prone PCR products (due to frame shifts and stop codons) to test whether our system can efficiently eliminate the false positive clones. As a consequence, we actually succeeded in demonstration of the viability of the two-step (or three-step) screening for screen the affinity-attenuated proteins (**Table 6** **and Table S5**), even though the system will still need to improve the accuracy rate to yield the target plasmid by two-step screening and to enable the screen of library with much smaller target frequency in the future.

We used the Z domain derived from *S. aureus* protein A as the candidate or known parental protein in the current study. As stated above, when the dimer of Z domain (ZZ) was used as the parental protein, the original ZZ proteins were identified as candidates (**Figs. S4 and S5**). However, controlling the expression levels (ratios) of the parental and candidate proteins should have allowed reliable selection (**Figs. S6 and S7**). In principle, the competitive approach binds candidate variants with similar affinities to the parental protein if the protein expression levels of ‘Y_1_’ and ‘Y_2_’ are identical (**Fig. 2A and B**). However, due to the low expression of proteins containing the lipidation motif and the low efficiency of lipidation modification, the relative amounts of the membrane-bound proteins (Y_1_) are generally lower than of the identical protein expressed in the cytosol (Y_2_). This highlights the unique feature of our system, namely, the ability to exclude protein mutants with the same affinity as the original proteins when using the monomeric Z variants (**Figs. 4** **–** **7**). Nevertheless, calibration of the parental and candidate protein expression levels is important to ensure a successful outcome using this screening method.

Our approach to create a desirable and ‘swingable’ screening methodology, which allows screening based on both affinity enhancement and affinity-attenuation, could prove innovative in research fields such as biochemistry and antibody mimetics, and could contribute to investigations of protein function or the development of new drugs.

## Supporting Information

Figure S1 **Localization of membrane-anchored Z protein with lipidation motif.** The pGK413-EGFP-ZWTmem-introduced BFG2118-ZK35Acyto yeast, which expressed the GFP-fused Z_WT_ with an artificial lipidation motif, was grown in SD-His, -Leu, -Ura medium at 30°C for 18 hours. The cell suspensions were observed with a confocal laser scanning microscope.
(TIFF)

Figure S2 **Flow diagram of two-step screening for screening of affinity-attenuated proteins.**
(TIF)

Figure S3 ***GFP*** **transcription assays of the engineered yeast strains using original Gγ recruitment system.** To enable two-step screening, the plasmids with additional restriction enzyme cleavage sites were constructed (pGK413-ZWTmem, pGK413-ZK35Amem, pGK413-ZI31Amem and pGK413-Z955mem). The plasmids were introduced into the engineered yeast lacking the expression of competitive proteins (BFG2118). Control yeast strain was transformed with pGK413 (Mock). The abilities for transducing the signal were identical to the strains with the equivalent plasmids without the restriction enzyme cleavage sites.
(TIFF)

Figure S4 ***GFP*** **transcription assays to test the selection of affinity-enhanced proteins.** (A) Flow cytometry analyses. (B) Fluorescence microscope observations. Fluorescence intensities and fluorescence micrographs of the engineered strains expressing cytosolic ZZ are shown.
(TIFF)

Figure S5 ***GFP*** **transcription assays to test the selection of affinity-attenuated proteins.** (A) Flow cytometry analyses. (B) Fluorescence microscope observations. Fluorescence intensities and fluorescence micrographs of the engineered strains expressing membrane-anchored ZZ are shown.
(TIFF)

Figure S6 ***GFP*** **transcription assays of the engineered yeast strains overexpressing cytosolic ZZ in the affinity-enhancement system.** To test whether cytosolic ZZ expression levels could reduce background signaling, ZZ as the ‘Y2’ parental protein was overexpressed using plasmid insertion in the affinity-enhanced system in addition to integration into the yeast chromosome. Using the multi-copy replication plasmid, the affinity-enhancement system never induced false-positive transcription of the *GFP* reporter gene.
(TIFF)

Figure S7 ***GFP*** **transcription assays of the engineered yeast strains overexpressing cytosolic ZZ in the affinity-attenuation system.** To test whether cytosolic ZZ expression levels affect background signaling, ZZ as the ‘Y_2_’ candidate protein was overexpressed using a multi-copy replication plasmid instead of the single-copy replication plasmid in the affinity-attenuation system. Using the multi-copy replication plasmid, the affinity-attenuation system never induced false-positive transcription of the *GFP* reporter gene.
(TIFF)

Table S1 **List of plasmids used in this study.**
(PDF)

Table S2 **List of primers used in this study.**
(PDF)

Table S3 **List of yeast transformants used to screen affinity-enhanced proteins.**
(PDF)

Table S4 **List of yeast transformants used to screen affinity-attenuated proteins**.
(PDF)

Table S5 **Composition of plasmid library.**
(PDF)

Table S6 **List of expressed proteins in the engineered yeast strains**.
(PDF)

Table S7 **List of yeast transformants for other supporting information.**
(PDF)
