# Supplementary figures and images for: Desired Alteration of Protein Affinities: Competitive Selection of Protein Variants Using Yeast Signal Transduction Machinery
Source: PLoS One. 2014 Sep 22;9(9):e108229. doi: 10.1371/journal.pone.0108229 (PMC4171513; doi:10.1371/journal.pone.0108229)

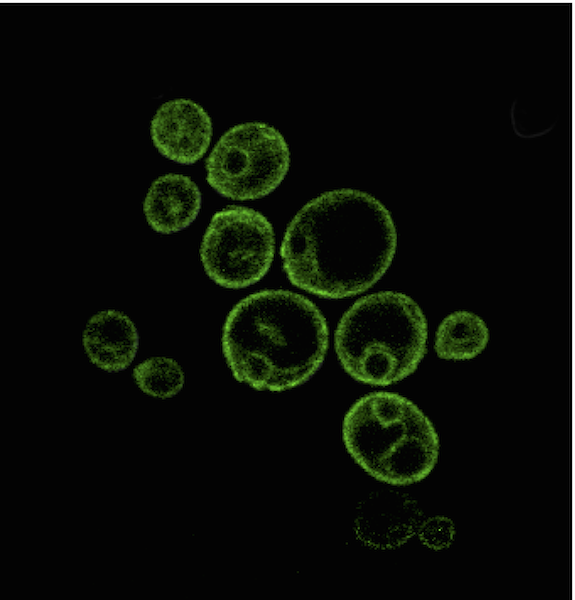

Supplement: Figure S1 — Localization of membrane-anchored Z protein with lipidation motif. The pGK413-EGFP-ZWTmem-introduced BFG2118-ZK35Acyto yeast, which expressed the GFP-fused ZWT with an artificial lipidation motif, was grown in SD-His, -Leu, -Ura medium at 30°C for 18 hours. The cell suspensions were observed with a confocal laser scanning microscope. (TIFF) [file pone.0108229.s001.tiff]

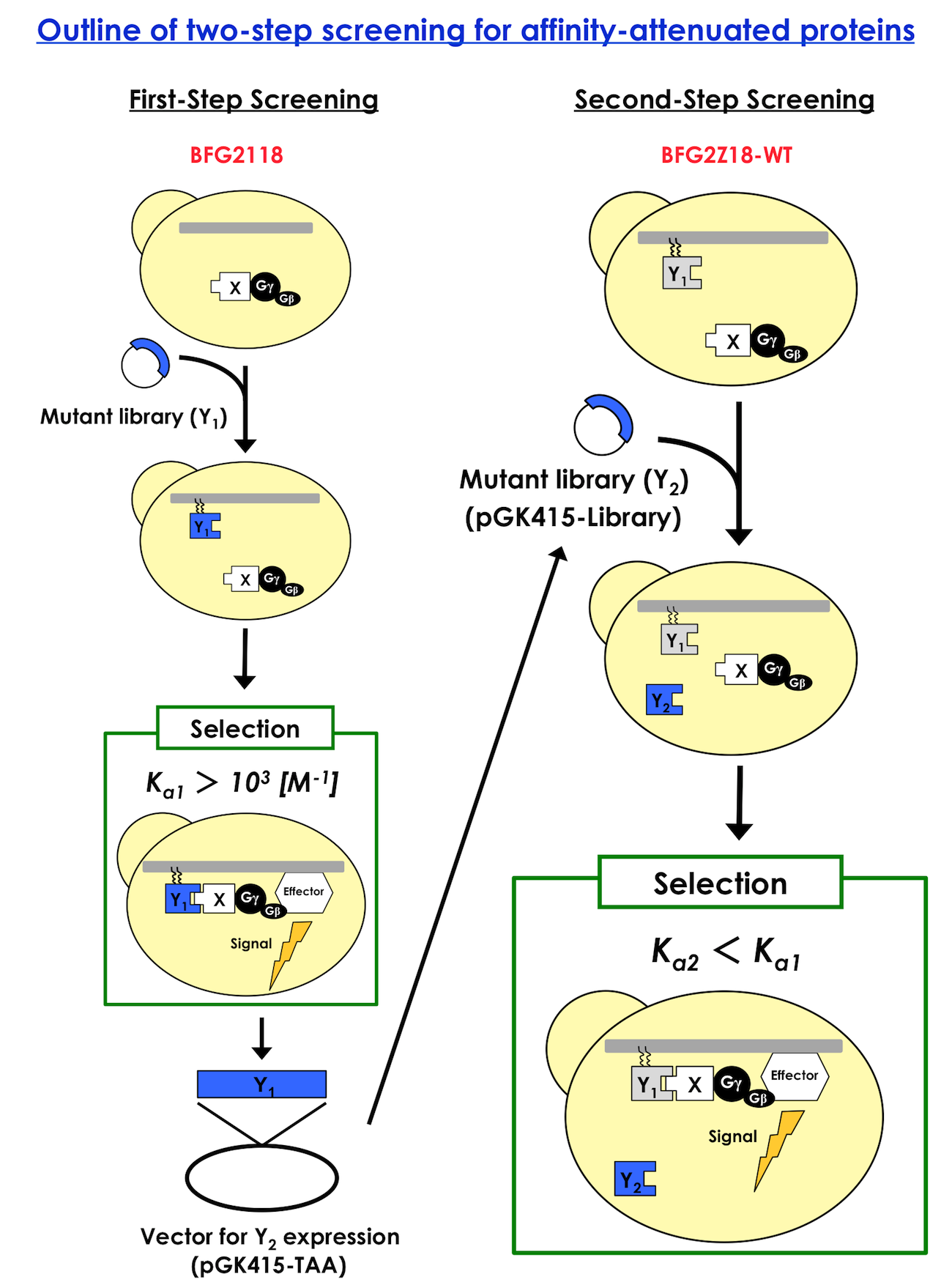

Supplement: Figure S2 — Flow diagram of two-step screening for screening of affinity-attenuated proteins. (TIF) [file pone.0108229.s002.tif]

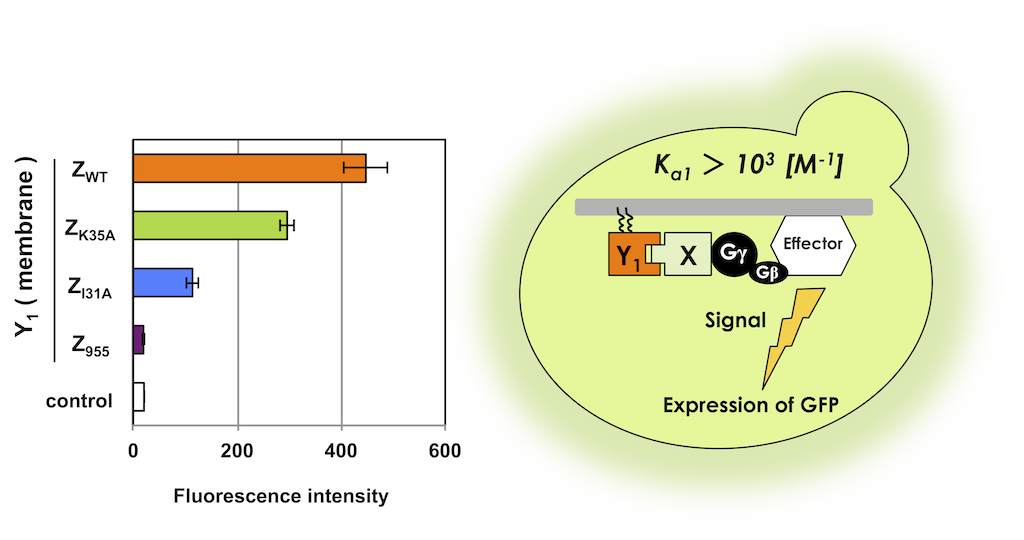

Supplement: Figure S3 — GFP transcription assays of the engineered yeast strains using original Gγ recruitment system. To enable two-step screening, the plasmids with additional restriction enzyme cleavage sites were constructed (pGK413-ZWTmem, pGK413-ZK35Amem, pGK413-ZI31Amem and pGK413-Z955mem). The plasmids were introduced into the engineered yeast lacking the expression of competitive proteins (BFG2118). Control yeast strain was transformed with pGK413 (Mock). The abilities for transducing the signal were identical to the strains with the equivalent plasmids without the restriction enzyme cleavage sites. (TIFF) [file pone.0108229.s003.tiff]

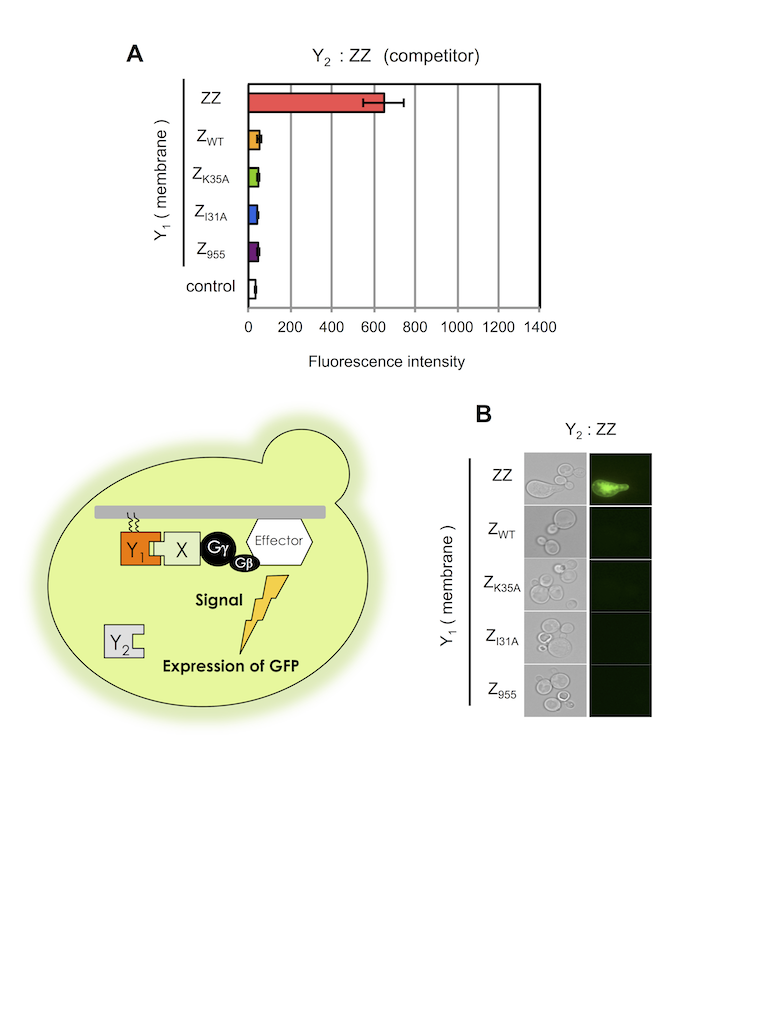

Supplement: Figure S4 — GFP transcription assays to test the selection of affinity-enhanced proteins. (A) Flow cytometry analyses. (B) Fluorescence microscope observations. Fluorescence intensities and fluorescence micrographs of the engineered strains expressing cytosolic ZZ are shown. (TIFF) [file pone.0108229.s004.tiff]

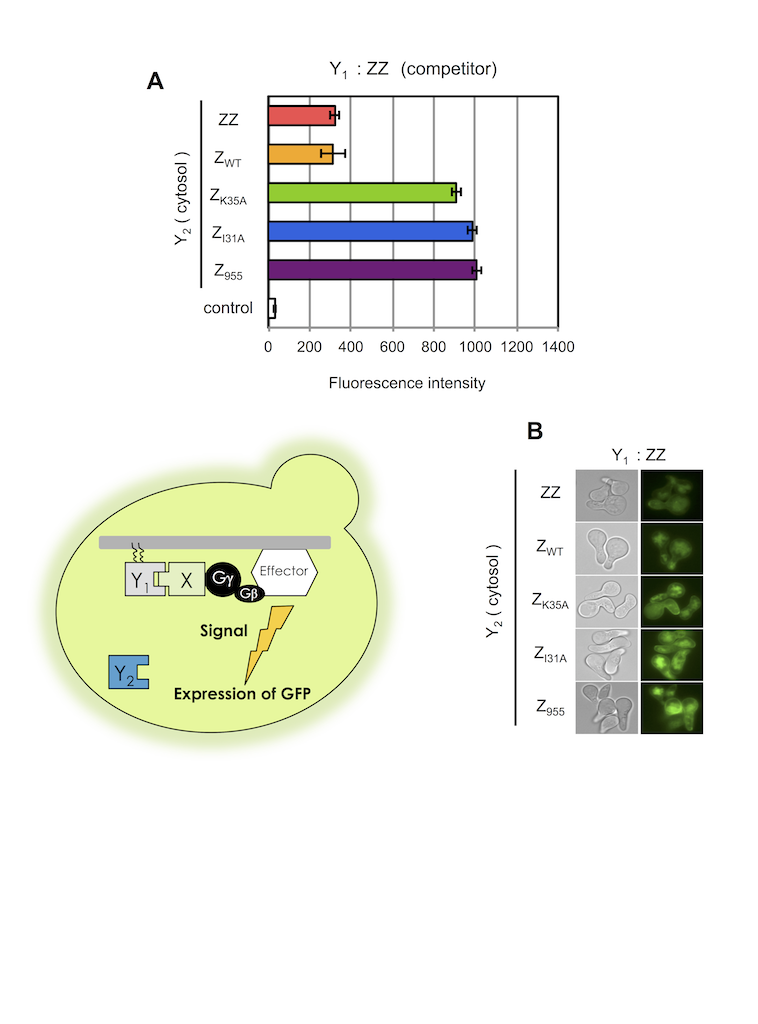

Supplement: Figure S5 — GFP transcription assays to test the selection of affinity-attenuated proteins. (A) Flow cytometry analyses. (B) Fluorescence microscope observations. Fluorescence intensities and fluorescence micrographs of the engineered strains expressing membrane-anchored ZZ are shown. (TIFF) [file pone.0108229.s005.tiff]

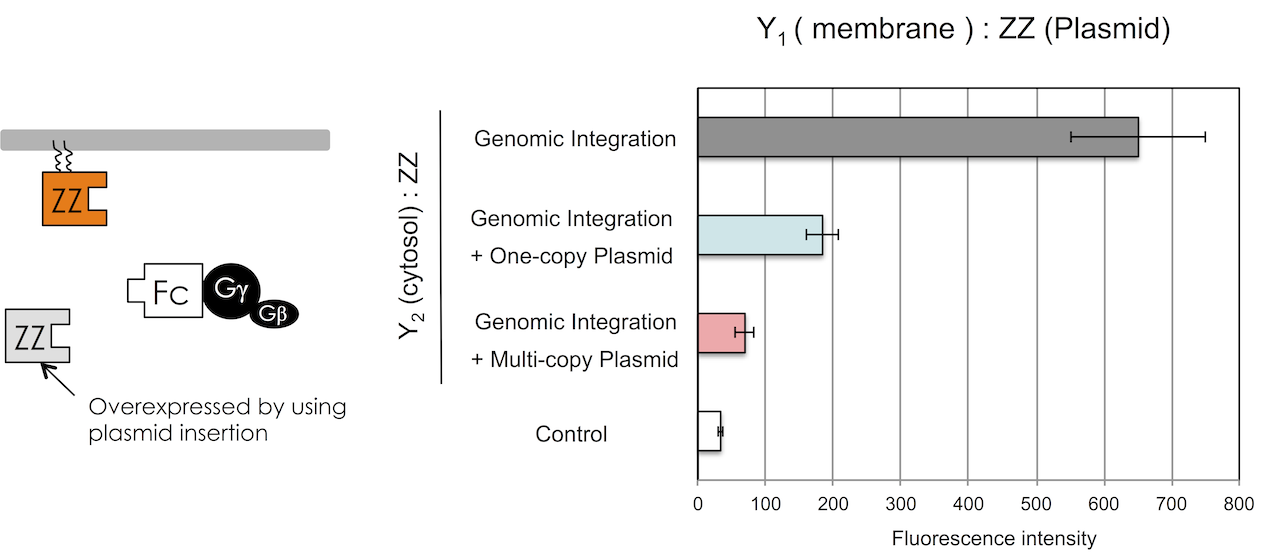

Supplement: Figure S6 — GFP transcription assays of the engineered yeast strains overexpressing cytosolic ZZ in the affinity-enhancement system. To test whether cytosolic ZZ expression levels could reduce background signaling, ZZ as the ‘Y2’ parental protein was overexpressed using plasmid insertion in the affinity-enhanced system in addition to integration into the yeast chromosome. Using the multi-copy replication plasmid, the affinity-enhancement system never induced false-positive transcription of the GFP reporter gene. (TIFF) [file pone.0108229.s006.tiff]

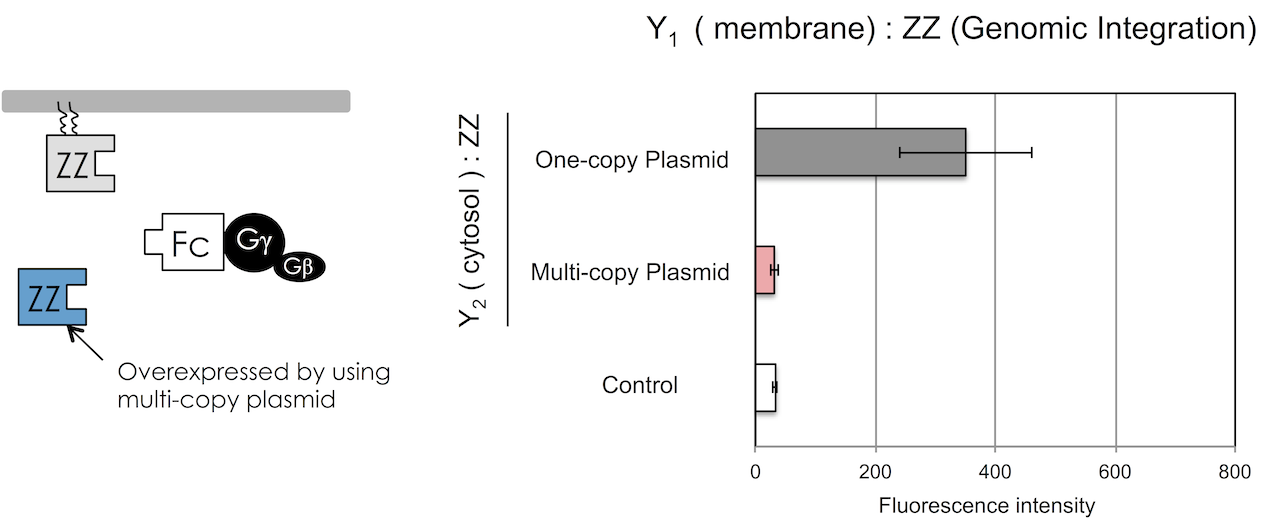

Supplement: Figure S7 — GFP transcription assays of the engineered yeast strains overexpressing cytosolic ZZ in the affinity-attenuation system. To test whether cytosolic ZZ expression levels affect background signaling, ZZ as the ‘Y2’ candidate protein was overexpressed using a multi-copy replication plasmid instead of the single-copy replication plasmid in the affinity-attenuation system. Using the multi-copy replication plasmid, the affinity-attenuation system never induced false-positive transcription of the GFP reporter gene. (TIFF) [file pone.0108229.s007.tiff]
