# Supplementary material for: Desired Alteration of Protein Affinities: Competitive Selection of Protein Variants Using Yeast Signal Transduction Machinery
Source: PLoS One. 2014 Sep 22;9(9):e108229. doi: 10.1371/journal.pone.0108229 (PMC4171513; doi:10.1371/journal.pone.0108229)
Supplement: Table S1 — List of plasmids used in this study. (PDF) [file pone.0108229.s008.pdf]

Table S1. List of plasmids used in this study.

Plasmids	Genotype	Reference source
**One-copy plasmids for expressing candidate Y_1 proteins (Z variants) on the membrane [for testing the affinity-enhancement system]**		
pGK413	Expression vector containing *PGK1* promoter, *CEN/ARS* single-copy origin and *HIS3* marker	Ishii J *et al.* (2009)
pGK-HsZZm	ZZ and C-terminus of Ste18 (9 a.a.) fusion expression, in pGK413	Present study
pGK-HsZm	Z_{WT} and C-terminus of Ste18 (9 a.a.) fusion expression, in pGK413	Present study
pGK-HsZK35Am	Z_{K35A} and C-terminus of Ste18 (9 a.a.) fusion expression, in pGK413	Present study
pGK-HsZI31Am	Z_{I31A} and C-terminus of Ste18 (9 a.a.) fusion expression, in pGK413	Present study
pGK-HsZ955m	Z_{955} and C-terminus of Ste18 (9 a.a.) fusion expression, in pGK413	Present study
**One-copy plasmids for expressing candidate Y_2 proteins (Z variants) in the cytosol [for testing the affinity-attenuation system]**		
pGK415	Expression vector containing *PGK1* promoter, *CEN/ARS* single-copy origin and *LEU2* marker	Ishii J *et al.* (2009)
pGK-LsZZc	ZZ expression, in pGK415	Present study
pGK-LsZWTc	Z_{WT} expression, in pGK415	Present study
pGK-LsZK35Ac	Z_{K35A} expression, in pGK415	Present study
pGK-LsZI31Ac	Z_{I31A} expression, in pGK415	Present study
pGK-LsZ955c	Z_{955} expression, in pGK415	Present study
**One-copy plasmids for expressing candidate Y_1 proteins (Z variants) on the membrane [for model screening]**		
pGK413-Ste18C	C-terminus of Ste18 (9 a.a.) expression, in pGK413	Present study
pGK413-ZWTmem	Z_{WT} expression, in pGK413-Ste18C	Present study
pGK413-ZK35Amem	Z_{K35A} expression, in pGK413-Ste18C	Present study
pGK413-ZI31Amem	Z_{I31A} expression, in pGK413-Ste18C	Present study
pGK413-Z955mem	Z_{955} expression, in pGK413-Ste18C	Present study
**One-copy plasmids for expressing candidate Y_2 proteins (Z variants) in the cytosol [for model screening]**		
pGK415-TAA	Add to stop codon after multi cloning site of pGK415	Present study

**High-copy plasmids for over-expressing ZZ as candidate Y_1 on the membrane or candidate Y_2 in the cytosol [for Supplementary Figures]**		
pGK425	Expression vector containing *PGK1* promoter, 2*μ* origin and *LEU2* marker	Ishii J *et al.* (2009)
pGK-LmZZc	ZZ and C-terminus of Ste18 (9 a.a.) fusion expression, in pGK425	Present study
pLMZ-ZZ-H	*LEU2-PGK5'-ZZ-PGK3'*-P_{HOP2} in pGK425	Present study
**One-copy plasmids for expressing EGFP- Z_{WT} expression on the membrane**		
pGK413-EGFP-N	EGFP expression in pGK413	Present study
pGK413-EGFP-ZWTmem	EGFP, Z_{WT} and C-terminus of Ste18 (9 a.a.) fusion, in pGK413	Present study
**Plasmids for constructing the yeast strains**		
pLMZ-WT-H	*LEU2-PGK5'*-Z_{WT}-*PGK3'*-P_{HOP2} in pGK425	Fukuda *et al.* (2010)
pLMZ-K35A-H	*LEU2-PGK5'*-Z_{K35A}-*PGK3'*-P_{HOP2} in pGK425	Fukuda *et al.* (2010)
pLMZ-I31A-H	*LEU2-PGK5'*-Z_{I31A}-*PGK3'*-P_{HOP2} in pGK425	Present study
