# Supplementary material for: Desired Alteration of Protein Affinities: Competitive Selection of Protein Variants Using Yeast Signal Transduction Machinery
Source: PLoS One. 2014 Sep 22;9(9):e108229. doi: 10.1371/journal.pone.0108229 (PMC4171513; doi:10.1371/journal.pone.0108229)
Supplement: Table S2 — List of primers used in this study. (PDF) [file pone.0108229.s009.pdf]

Table S2. List of primers used in this study.

Number	Primer name	Sequence (5' to 3')
1	SalI-EZZ-fw	ttttgtcgacgcgcaacacgatgaagccgt
2	BamHI-Ste18c30-Z(131-174)-rv	aaaaggatccttacataagcgtacaacaaacactatttgatttcggcgcctgagcatcatttagctttttagcttctgctaaaatttt -gtcgacgcgcaacacgatgaagccgt
3	SalI-atg-FlagEZ(1-20)-fw	ttttgtcgacatggactacaaggatgacgatgacaaggcgcaacacgatgaagccgtagacaacaaattcaacaa
4	BamHI-tag-Z(154-174)-rv	tttttggatccctatttcggcgcctgagcatca
5	XmaI-Ste18C-PGK3'-fw	aaaacccgggtcaaatagtgtttgttgtacgcttatgtaaaaagatgccgatttgggcgcgaatc
6	NotI-PGK3'end-rv	ttttgcggccgcagctttaacgaacgcagaattt
7	SalI-atg-EZ(1-20)-fw	ttttgtcgacatggcgcaacacgatgaagccgtagacaacaaattcaacaa
8	BamHI-Z(160-174)-rv	ttttggatcctttcggcgcctgagc
9	BamHI-TAA-XmaI-PGK3'-fw	aaaaggatcctaacccgggtctagagaattcagatct
10	SalI-start-EGFP-fw	aaaagtcgacatggtgagcaagggc
11	BamHI-EGFP-rv	ttttggatcccttgtacagctcgtcca
12	BamHI-E domain-fw	aaaaggatccgcgcaacacgatgaagcc
13	XmaI-end-Ste18C-rv	ttttcccgggttacataagcgtacaacaaa
14	Hop2p 150-200-LEU2-fw	atacaattaattgacatcagcagacagcaaatgcacttgatatacgcagctcgactacgtcgtaaggccgt
15	Hop2p 800-rv	atctttcaaatagagcctgg
16	pGK413-fw	gcatcagagcagattgtactgagagtgcaccataaattcc
17	pGK413-rv	cgcaaaccgcctctccccgcgcgttggccgattcattaat
18	pGK415-fw	cttacctgtattcctttactatcctcctttttctccttct
19	pGK415-rv	ggaattgtgagcggataacaatttcacacaggaaacagct
