# Supplementary material for: Desired Alteration of Protein Affinities: Competitive Selection of Protein Variants Using Yeast Signal Transduction Machinery
Source: PLoS One. 2014 Sep 22;9(9):e108229. doi: 10.1371/journal.pone.0108229 (PMC4171513; doi:10.1371/journal.pone.0108229)
Supplement: Table S3 — List of yeast transformants used to screen affinity-enhanced proteins. (PDF) [file pone.0108229.s010.pdf]

Table S3. List of yeast transformants used to screen affinity-enhanced proteins.

Transformants	Parent Y_2 (cytosol)	Candidate Y_1 (membrane)	Target X	Figures
**[BFG2118-ZWTcyto]**				
BFG2118-ZWTcyto + (pGK-Hs-ZZm)	Z_{WT} (Gen)	ZZ_{mem} (Pla)	Fc	Figs 4a,d and 5a
BFG2118-ZWTcyto + (pGK-Hs-ZWTm)	Z_{WT} (Gen)	$Z_{WT,mem}$ (Pla)	Fc	Figs 4a,d and 5a
BFG2118-ZWTcyto + (pGK-Hs-ZK35Am)	Z_{WT} (Gen)	$Z_{K35A,mem}$ (Pla)	Fc	Figs 4a,d and 5a
BFG2118-ZWTcyto + (pGK-Hs-ZI31Am)	Z_{WT} (Gen)	$Z_{I31A,mem}$ (Pla)	Fc	Figs 4a,d and 5a
BFG2118-ZWTcyto + (pGK-Hs-Z955m)	Z_{WT} (Gen)	$Z_{955,mem}$ (Pla)	Fc	Figs 4a,d and 5a
BFG2118-ZWTcyto + (pGK413) [control]	Z_{WT} (Gen)	– (Pla)	Fc	Figs 4a,d and 5a
**[BFG2118-ZK35Acyto]**				
BFG2118-ZK35Acyto + (pGK-Hs-ZZm)	Z_{K35A} (Gen)	ZZ_{mem} (Pla)	Fc	Figs 4b,e and 5b
BFG2118-ZK35Acyto + (pGK-Hs-ZWTm)	Z_{K35A} (Gen)	$Z_{WT,mem}$ (Pla)	Fc	Figs 4b,e and 5b
BFG2118-ZK35Acyto + (pGK-Hs-ZK35Am)	Z_{K35A} (Gen)	$Z_{K35A,mem}$ (Pla)	Fc	Figs 4b,e and 5b
BFG2118-ZK35Acyto + (pGK-Hs-ZI31Am)	Z_{K35A} (Gen)	$Z_{I31A,mem}$ (Pla)	Fc	Figs 4b,e and 5b
BFG2118-ZK35Acyto + (pGK-Hs-Z955m)	Z_{K35A} (Gen)	$Z_{955,mem}$ (Pla)	Fc	Figs 4b,e and 5b
BFG2118-ZK35Acyto + (pGK413) [control]	Z_{K35A} (Gen)	– (Pla)	Fc	Figs 4b,e and 5b
**[BFG2118-ZI31Acyto]**				
BFG2118-ZI31Acyto + (pGK-Hs-ZZm)	Z_{I31A} (Gen)	ZZ_{mem} (Pla)	Fc	Figs 4c,f and 5c
BFG2118-ZI31Acyto + (pGK-Hs-ZWTm)	Z_{I31A} (Gen)	$Z_{WT,mem}$ (Pla)	Fc	Figs 4c,f and 5c
BFG2118-ZI31Acyto + (pGK-Hs-ZK35Am)	Z_{I31A} (Gen)	$Z_{K35A,mem}$ (Pla)	Fc	Figs 4c,f and 5c
BFG2118-ZI31Acyto + (pGK-Hs-ZI31Am)	Z_{I31A} (Gen)	$Z_{I31A,mem}$ (Pla)	Fc	Figs 4c,f and 5c
BFG2118-ZI31Acyto + (pGK-Hs-Z955m)	Z_{I31A} (Gen)	$Z_{955,mem}$ (Pla)	Fc	Figs 4c,f and 5c
BFG2118-ZI31Acyto + (pGK413) [control]	Z_{I31A} (Gen)	– (Pla)	Fc	Figs 4c,f and 5c
**[BFG2118-ZZcyto]**				
BFG2118-ZZcyto + (pGK-Hs-ZZm)	ZZ (Gen)	ZZ_{mem} (Pla)	Fc	Supplementary Figs S3a,b and S5
BFG2118-ZZcyto + (pGK-Hs-ZWTm)	ZZ (Gen)	$Z_{WT,mem}$ (Pla)	Fc	Supplementary Fig. S3a,b
BFG2118-ZZcyto + (pGK-Hs-ZK35Am)	ZZ (Gen)	$Z_{K35A,mem}$ (Pla)	Fc	Supplementary Fig. S3a,b
BFG2118-ZZcyto + (pGK-Hs-ZI31Am)	ZZ (Gen)	$Z_{955,mem}$ (Pla)	Fc	Supplementary Fig. S3a,b
BFG2118-ZZcyto + (pGK413) [control]	ZZ (Gen)	– (Pla)	Fc	Supplementary Figs S3a,b and S5
BFG2118-ZZcyto + (pGK-Ls-ZZc) + (pGK-Hs-ZZm)	ZZ (Gen) + ZZ (Pla)	ZZ_{mem} (Pla)	Fc	Supplementary Fig. S5
BFG2118-ZZcyto + (pGK-Lm-ZZc) + (pGK-Hs-ZZm)	ZZ (Gen) + ZZ (Hi-Pla)	ZZ_{mem} (Pla)	Fc	Supplementary Fig. S5

* “Gen” means Genome expression. “Pla” means One-copy Plasmid expression. “Hi-Pla” means High-copy Plasmid expression.
