# Supplementary material for: Desired Alteration of Protein Affinities: Competitive Selection of Protein Variants Using Yeast Signal Transduction Machinery
Source: PLoS One. 2014 Sep 22;9(9):e108229. doi: 10.1371/journal.pone.0108229 (PMC4171513; doi:10.1371/journal.pone.0108229)
Supplement: Table S4 — List of yeast transformants used to screen affinity-attenuated proteins. (PDF) [file pone.0108229.s011.pdf]

Table S4. List of yeast transformants used to screen affinity-attenuated proteins.

Transformants	Parent Y_1 (membrane)	Candidate Y_2 (cytosol)	Target X	Figures
**[BFG2Z18-WT]**				
BFG2Z18-WT + (pGK-Ls-ZZc)	$Z_{WT,mem}$ (Gen)	ZZ (Pla)	Fc	Figs 6a,d and 7a
BFG2Z18-WT + (pGK-Ls-ZWTc)	$Z_{WT,mem}$ (Gen)	Z_{WT} (Pla)	Fc	Figs 6a,d and 7a
BFG2Z18-WT + (pGK-Ls-ZK35Ac)	$Z_{WT,mem}$ (Gen)	Z_{K35A} (Pla)	Fc	Figs 6a,d and 7a
BFG2Z18-WT + (pGK-Ls-ZI31Ac)	$Z_{WT,mem}$ (Gen)	Z_{I31A} (Pla)	Fc	Figs 6a,d and 7a
BFG2Z18-WT + (pGK-Ls-Z955Ac)	$Z_{WT,mem}$ (Gen)	Z_{955} (Pla)	Fc	Figs 6a,d and 7a
BFG2118+ (pGK415) [control]	–	– (Pla)	Fc	Figs 6a,d and 7a
**[BFG2Z18-K35A]**				
BFG2Z18-K35A + (pGK-Ls-ZZc)	$Z_{K35A,mem}$ (Gen)	ZZ (Pla)	Fc	Figs 6b,e and 7b
BFG2Z18-K35A + (pGK-Ls-ZWTc)	$Z_{K35A,mem}$ (Gen)	Z_{WT} (Pla)	Fc	Figs 6b,e and 7b
BFG2Z18-K35A + (pGK-Ls-ZK35Ac)	$Z_{K35A,mem}$ (Gen)	Z_{K35A} (Pla)	Fc	Figs 6b,e and 7b
BFG2Z18-K35A + (pGK-Ls-ZI31Ac)	$Z_{K35A,mem}$ (Gen)	Z_{I31A} (Pla)	Fc	Figs 6b,e and 7b
BFG2Z18-K35A + (pGK-Ls-Z955c)	$Z_{K35A,mem}$ (Gen)	Z_{955} (Pla)	Fc	Figs 6b,e and 7b
BFG2118 + (pGK415) [control]	–	– (Pla)	Fc	Figs 6b,e and 7b
**[BFG2Z18-I31A]**				
BFG2Z18-I31A + (pGK-Ls-ZZc)	$Z_{I31A,mem}$ (Gen)	ZZ (Pla)	Fc	Figs 6c,f and 7c
BFG2Z18-I31A + (pGK-Ls-ZWTc)	$Z_{I31A,mem}$ (Gen)	Z_{WT} (Pla)	Fc	Figs 6c,f and 7c
BFG2Z18-I31A + (pGK-Ls-ZK35Ac)	$Z_{I31A,mem}$ (Gen)	Z_{K35A} (Pla)	Fc	Figs 6c,f and 7c
BFG2Z18-I31A + (pGK-Ls-ZI31Ac)	$Z_{I31A,mem}$ (Gen)	Z_{I31A} (Pla)	Fc	Figs 6c,f and 7c
BFG2Z18-I31A + (pGK-Ls-Z955)	$Z_{I31A,mem}$ (Gen)	Z_{955} (Pla)	Fc	Figs 6c,f and 7c
BFG2118 + (pGK415) [control]	–	– (Pla)	Fc	Figs 6c,f and 7c
**[BZFG2118]**				
BZFG2118 + (pGK-Ls-ZZc)	ZZ (Gen)	ZZ (Pla)	Fc	Supplementary Figs S4a,b and S6
BZFG2118 + (pGK-Ls-ZWTc)	ZZ (Gen)	Z_{WT} (Pla)	Fc	Supplementary Fig. S4a,b
BZFG2118 + (pGK-Ls-ZK35Ac)	ZZ (Gen)	Z_{K35A} (Pla)	Fc	Supplementary Fig. S4a,b
BZFG2118 + (pGK-Ls-ZI31Ac)	ZZ (Gen)	Z_{I31A} (Pla)	Fc	Supplementary Fig. S4a,b
BZFG2118 + (pGK-Ls-Z955c)	ZZ (Gen)	Z_{955} (Pla)	Fc	Supplementary Fig. S4a,b
BFG2118 + (pGK415) [control]	–	– (Pla)	Fc	Supplementary Figs S4a,b and S6
BZFG2118 + (pGK-Lm-ZZc)	ZZ (Gen)	ZZ (Hi-Pla)	Fc	Supplementary Fig S6

* "Gen" means Genome expression. "Pla" means One-copy Plasmid expression. "Hi-Pla" means High-copy Plasmid expression.
