# Supplementary material for: Desired Alteration of Protein Affinities: Competitive Selection of Protein Variants Using Yeast Signal Transduction Machinery
Source: PLoS One. 2014 Sep 22;9(9):e108229. doi: 10.1371/journal.pone.0108229 (PMC4171513; doi:10.1371/journal.pone.0108229)
Supplement: Table S5 — Composition of plasmid library. (PDF) [file pone.0108229.s012.pdf]

Table S5. Composition of plasmid library.

Model library for testing the affinity-enhancement system

No.	Model library composition	Initial ratio of target plasmid (Z_{WT})
1	Z_{WT} : Z_{K35A} : Z_{I31A} : Z_{955} : Mock (pGK413) = 10 : 10 : 25 : 25 : 30	10.0%
2	Z_{WT} : Z_{K35A} : Z_{I31A} : Z_{955} : Mock (pGK413) = 1 : 10 : 30 : 30 : 30	1.0%
3	Z_{WT} : Z_{K35A} : Z_{I31A} : Z_{955} : Mock (pGK413) = 0.5 : 1 : 35 : 35 : 30	0.5%
4	Z_{WT} : Z_{K35A} : Z_{I31A} : Z_{955} : Mock (pGK413) = 0.1 : 1 : 35 : 35 : 30	0.1%

Model library for testing the affinity-attenuation system

No.	Model library composition	Initial ratio of target plasmid (Z_{K35A} + Z_{I31A})
1	Z_{WT} : Z_{K35A} : Z_{I31A} : Z_{955} : Mock (pGK413) = 1 : 1 : 1 : 70 : 30	2.0%
2	Z_{WT} : Z_{K35A} : Z_{I31A} : Z_{955} : Mock (pGK413) = 0.1 : 0.1 : 0.1 : 55 : 45	0.2%
