# Supplementary material for: Desired Alteration of Protein Affinities: Competitive Selection of Protein Variants Using Yeast Signal Transduction Machinery
Source: PLoS One. 2014 Sep 22;9(9):e108229. doi: 10.1371/journal.pone.0108229 (PMC4171513; doi:10.1371/journal.pone.0108229)
Supplement: Table S6 — List of expressed proteins in the engineered yeast strains. (PDF) [file pone.0108229.s013.pdf]

Table S6. List of expressed proteins in the engineered yeast strains.

Strain	$G\gamma_{cyto}$-fused target protein (X)	Membrane-anchored parental protein (Y_1)	Cytosolic parental protein (Y_2)
**Control**			
BFG2118	$G\gamma_{cyto}$-Fc	–	–
**Yeast strains for screening affinity-enhanced proteins**			
BFG2118-ZZcyto	$G\gamma_{cyto}$-Fc	–	ZZ
BFG2118-ZWTcyto	$G\gamma_{cyto}$-Fc	–	Z_{WT}
BFG2118-ZK35Acyto	$G\gamma_{cyto}$-Fc	–	Z_{K35A}
BFG2118-ZI31Acyto	$G\gamma_{cyto}$-Fc	–	Z_{I31A}
**Yeast strains for screening affinity-attenuated proteins**			
BZFG2118	$G\gamma_{cyto}$-Fc	ZZ_{mem}	–
BFG2Z18-WT	$G\gamma_{cyto}$-Fc	$Z_{WT,mem}$	–
BFG2Z18-K35A	$G\gamma_{cyto}$-Fc	$Z_{K35A,mem}$	–
BFG2Z18-I31A	$G\gamma_{cyto}$-Fc	$Z_{I31A,mem}$	–
