# Supplementary material for: Desired Alteration of Protein Affinities: Competitive Selection of Protein Variants Using Yeast Signal Transduction Machinery
Source: PLoS One. 2014 Sep 22;9(9):e108229. doi: 10.1371/journal.pone.0108229 (PMC4171513; doi:10.1371/journal.pone.0108229)
Supplement: Table S7 — List of yeast transformants for other supporting information. (PDF) [file pone.0108229.s014.pdf]

Table S7. List of yeast transformants for other supporting information.

Transformants	Y_1 (membrane)	Y_2 (cytosol)	X (Target)	Figures
**[BFG2118-ZK35Acyto]**				
BFG2118-ZK35Acyto + (pGK413-EGFP-ZWTmwm)	EGFP-$Z_{WT,mem}$ (Pla)	Z_{K35A} (Gen)	Fc	Supplementary Fig S1

Transformants	Candidate Y_1 (membrane)	Target X	Figures
**[BFG2118-ZWTcyto]**			
BFG2118 + (pGK413-ZWTmem)	$Z_{WT,mem}$ (Pla)	Fc	Supplementary Fig S7
BFG2118 + (pGK413-ZK35Amem)	$Z_{K35A,mem}$ (Pla)	Fc	Supplementary Fig S7
BFG2118 + (pGK413-ZI31Amem)	$Z_{I31A,mem}$ (Pla)	Fc	Supplementary Fig S7
BFG2118 + (pGK413-Z955mem)	$Z_{955,mem}$ (Pla)	Fc	Supplementary Fig S7
BFG2118 + (pGK413) [control]	– (Pla)	Fc	Supplementary Fig S7

* “Gen” means Genome expression. “Pla” means One-copy Plasmid expression.
